# Mutual Solubilities between Ethylene Glycol and Organic Diluents: Gas Chromatography and NMR

**DOI:** 10.3390/molecules28135121

**Published:** 2023-06-29

**Authors:** Maria Atanassova, Vanya Kurteva

**Affiliations:** 1 Department of General and Inorganic Chemistry, University of Chemical Technology and Metallurgy, 8 Kliment Okhridski Blvd., 1756 Sofia, Bulgaria; 2 Institute of Organic Chemistry with Centre of Phytochemistry, Bulgarian Academy of Sciences, Acad. G. Bonchev Street, Block 9, 1113 Sofia, Bulgaria

**Keywords:** phase equilibria, solubility, ethylene glycol, organic diluents

## Abstract

In this work, the mutual solubilities of sets of organic diluents (CHCl_3_, C_6_H_6_, C_2_H_4_Cl_2_, CCl_4_, C_6_H_12_, and n-hexane) with the organic compound ethylene glycol are investigated via gas chromatography (GC). The experimental data measured for these binary organic systems are used to adjust the future nonaqueous systems for the solvent extraction of various metals with ligands. The obtained results showed that the solubility of ethylene glycol decreased in the order CHCl_3_ > C_6_H_6_ > C_2_H_4_Cl_2_ > CCl_4_(0%) ≈ C_6_H_12_ ≈ n-hexane. On the other hand, the solubility of the tested traditional organic diluents in ethylene glycol decreased in the following order: C_6_H_6_ > CHCl_3_ > C_2_H_4_Cl_2_ > n-hexane > C_6_H_12_ > CCl_4_. ^1^H NMR was also used as an analytic method in order to compare the obtained results for the samples showing significant solubility only, including an additional study with 1,2- or 1,3-propanediol. The enhanced solubility of the C_6_H_6_ compound in ethylene glycol was identified here as critical due to the GC technique, which will be without future consequences in chemical technology. Therefore, it was found that the best molecular diluent for the recovery of metals among the tested ones is C_6_H_12_, with a green protocol as the new paradigm, replacing the aqueous phase with another nonaqueous phase, i.e., a second organic diluent.

## 1. Introduction

Nowadays, one important aspect of scientific efforts to develop greener separations is the application and evaluation of innovative solvent systems, usually with no drawbacks associated with their conventional organic counterparts [1,2,3,4]. Among various separation methods, somehow, liquid–liquid extraction should always clearly focus more attention on environmental “friendliness”; it suffers from one very significant weakness, i.e., the need for water-immiscible organic diluents [5,6,7,8]. In general, during the liquid–liquid extraction processes of metallic species—usually with ligands—the two phases should be immiscible as they can be mixed together in all proportions to form two separate appearances at ambient temperature and pressure, enacting the lucid criterion [9,10]. Therefore, solvent extraction circuits should have only two phases: traditionally aqueous and organic. It should be noted that a regular diluent screening is often less time-consuming and less expensive, although the prediction of diluent effects is a challenging task [11,12,13,14,15,16]. Furthermore, the large catalogue of molecular diluents available for experimental work is one of the delightful aspects of solvent extraction chemistry (and probably in other fields of chemistry) [17]. There are a wide range to choose from, and each has its special applications, where it, in some way or another, outshines the rest [18]. Another consideration when selecting an extraction diluent is its density. In other words, diluents that are denser than water will form the lower layer of the pair when mixing together, while diluents that are less dense than water will form the upper layer or “float” on water. Therefore, water would form the top layer in a water–chloroform pair; the density of chloroform is 1.4892 g∙cm^−3^ at 20 °C. However, it should only contain carbon, hydrogen, nitrogen, and oxygen to be totally incinerable [5]—if possible, of course. Although the two diluents (usually inorganic and organic water) may form two visibly distinct phases when mixing together, they are often somewhat soluble in each other and will, in fact, become somehow mutually saturated. As one exemplification, the solubility of water in several diluents in mol·dm^−3^ unit (often abbreviated as M) decreases in the order: 1,2-dichloroethane (0.1262) > CHCl_3_ (0.0738) > C_6_H_6_ (0.0349) > toluene (0.027) ≈ *p*-xylene (0.021) > CCl_4_ (0.0087) > C_6_H_12_ (0.0029) [19]. On the other hand, it is known that the solubility of water in heptane is extremely low (0.0003% *w*/*w* or 3 mg L^−1^, or 3 × 10^−5^ M) [19,20]. Meanwhile, the solubility of water in organic diluents is generally several orders of magnitude higher than its solubility in water. As a result, among the many environmentally benign issues today is the viability of the suitable use of some organic liquid compounds as replacements of water in chemical technology. As a whole, applications of ethylene glycol compound have been summarized in a number of excellent investigations with regard to its potential use in separation processes [21,22,23,24,25,26,27]. However, attention must be paid to the chemical nature, toxicity, and biodegradation of such compounds apropos their solubility in water, which poses serious concern regarding their possible entry into ecosystems. For the two-phase liquid system, the question arises about how much of each of the pure liquid components dissolves in the other at equilibrium at a given temperature and pressure [28,29,30,31]. However, the critical aspect here is the mutual solubility between the two immiscible organic liquids, so the knowledge of the mixing behavior is of great relevance for the purpose of designing innovative nonaqueous solvent systems. Under this green scheme, liquid–liquid metal extraction is immediately looking for an ecological revivification today. As an example, some recent studies have addressed the use of novel nanocarriers like cationic green nanoemulsion as a promising strategy to treat and control water contamination as they are simple, rapid, economic, scalable, and have a high-efficiency mechanism compared to the well-known conventional methods [32,33].

The possibility of controlling solubility properties by adequately manipulating the nature of the two organic liquids allows for the design of such solvent systems in the fluid mixtures to exhibit selective characteristics as excellent alternative green diluents for extraction purposes for various metal ions [34,35,36,37,38,39]. In the last decade, these observations have raised additional research concerns regarding the viability of ionic liquid (IL) compounds like “drop-in replacements” for traditional organic diluents [5,6,7,22,28]. Unfortunately, under certain experimental conditions, their aqueous solubility may be significant, with an appreciable loss of IL cation or anion entities, which clearly poses a problem [5,34,35,36,37,38,39]. Moreover, one can see that the degree of solubility of the highly hydrophobic ILs in water is much smaller as compared to typical nonpolar aprotic diluents (CHCl_3_) [5]. Thus, the question should be addressed through a simple investigation of the phase equilibrium and interactions between two liquid compounds.

In this study, the influences of the chemical nature of several typical organic diluents on their solubility in another organic diluent like ethylene glycol, for example, are presented. In fact, eight liquid–liquid binary systems were investigated using gas chromatography. NMR spectroscopy was also used in the experimental cases with relatively high solubility in ethylene glycol, with a view to establish some approximate correlation. The requirement for conducting this research and the main motivation were the possible application of such nonaqueous systems for the solvent extraction of various metals in the future, in order to tweak the concerns connected with more green chemistry in chemical technology. Such studies are of interest in the field of solvent extraction chemistry of metals, but also from a wider perspective, in order to predict their potential eco-impact and to overcome their leaching drawback by adjusting the chemical composition of the solvent systems in use, employing a new original medium.

## 2. Results and Discussion

### 2.1. Studies Based on Gas Chromatography

**Figure 1:**
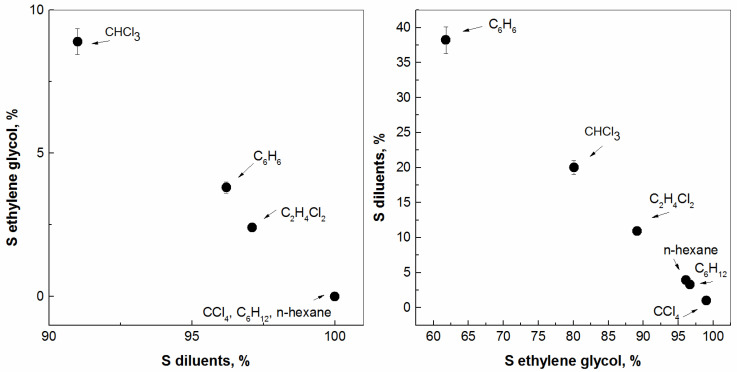
Solubility (S) of ethylene glycol in several organic diluents (**left**) as well as solubility of these organic diluents in ethylene glycol (**right**).

In this research investigation, we assessed the fundamental aspects of the mutual miscibility of diluents by studying the mixing of two potential organic liquid candidates for future nonaqueous solvent extraction processes. In the present study, several organic diluents with a different chemical nature were selected, i.e., polar and nonpolar, with a view to investigate their mutual solubility with ethylene glycol (EtG). As one would expect, the first indication of mutual solubility is a volume change in two liquid layers that are in contact with each other, but it is good, from a scientific point of view, to quantify this more precisely if possible. On the other hand, it does not automatically hold that the mutual solubility is the same in the two liquid compounds. As a whole, the transfer of diluent molecules from one phase to the other is more often not equal, for example, taking into account the obtained data in the literature with the water medium [5]. The data obtained from the analysis of the samples using gas chromatography are presented in Figure 1. Thus, the data for the six fluid systems obtained in both directions are summarized: organic diluent → ethylene glycol as well as ethylene glycol → organic diluent. In general, it can be concluded that the solubility of the compound ethylene glycol in the studied organic diluents was negligible, at slightly below 5%, with one exception, of course, namely chloroform. For the group of organic diluents such as CCl_4_, C_6_H_12_, and C_2_H_4_Cl_2_, the calculated percentages were ca. 0%. However, this was not the case concerning the transfer of organic diluent → ethylene glycol. As can be seen from Figure 1, the obtained values of 1–3.9% were precisely calculated for these organic diluents, followed by C_2_H_4_Cl_2_ and the extremely high solubility observed for CHCl_3_ and C_6_H_6_. Therefore, the following order of decreasing solubility of organic diluents in ethylene glycol can be proposed: C_6_H_6_ > CHCl_3_ > C_2_H_4_Cl_2_ > n-hexane > C_6_H_12_ > CCl_4_. However, why is the nonpolar benzene compound on the other side of the row and before the polar diluents in this order? In the case of benzene in liquid–liquid extraction, the interaction with the employed extractants would be primarily due to π−π interactions between phenyls, that are likely weaker than most hydrogen bond interactions [40]. Herein, EtG may form H-bonds with chloroform and 1,2-dichloroethane, between the hydrogen-donating OH group of EtG and hydrogen-accepting Cl−atoms of the diluent in use. For the mentioned diluents, the chlorine atoms withdraw the electron density of the neighboring atoms, which weakens the adjacent C−H bonds. Of course, the CHCl_3_ protons would give much stronger H−bonds with the EtG O−atoms by favoring a stronger hydrogen bond network [41], compared to 1,2-dichloroethane. In fact, the diluent gains the ability to enter into hydrogen bonding with the extractant or the extracted metal complex, in accordance with the following trend described by Kolarik [42]: inert diluents > n-donor > π-donor > acceptor. Any organic compound having a vapour pressure of 0.01 kPa or more at 20 °C is volatile—a VOC. In fact, the modern chemical trend is generally against diluents containing aromatic components, e.g., C_6_H_6_, because of their lower boiling and flash points, higher volatility and toxicity, carcinogenic properties, greater flammability, and the need to limit carbon emissions in compliance with the applicable environmental legislation, including higher aqueous-phase solubility. Nevertheless, the efficiency of solvent extraction carried out with aromatic and aliphatic hydrocarbons diluents is more important than with chlorinated hydrocarbons, because of the interest on an industrial scale. 

**Figure 2:**
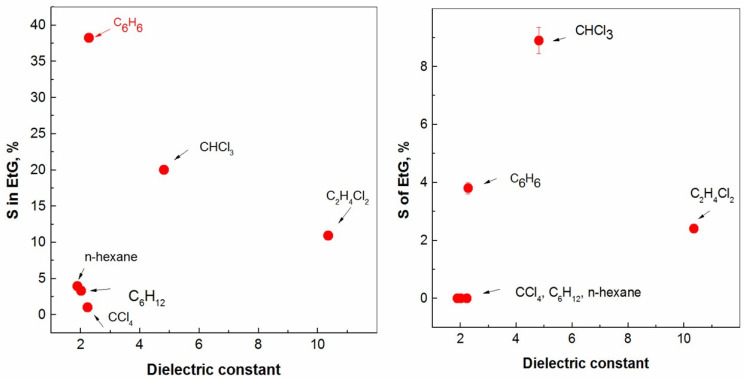
Solubility of organic diluents in ethylene glycol vs. dielectric constant (**left**) as well as solubility of ethylene glycol in organic diluents vs. diluents’ dielectric constant (**right**).

The next figure, Figure 2, shows the dependencies obtained from the solubility data relative to the dielectric constant of each diluent. If, for the two polar diluents containing chlorine, the increase in solubility can be explained by the characteristic relatively high dielectric constants, benzene remains, however, outside of this framework. The data in Figure 2 (right) show only CHCl_3_ as noteworthy; the others are within 5%, i.e., not significant enough to be commented upon at all. It is a known fact that the chemical nature of diluents usually has some significant influence on the solvent extraction equilibria [10]. In other words, the distribution ratios usually increase with the polarity of the organic phase [13]. In general, the equilibrium constants (K_ex_) of the extracted complexes are evaluated and correlate with the dielectric constants and dipole moments of the investigated diluents [15]. Moreover, the inner synergistic effect increases with a decreasing organic diluent polarity [9,16].

**Figure 3:**
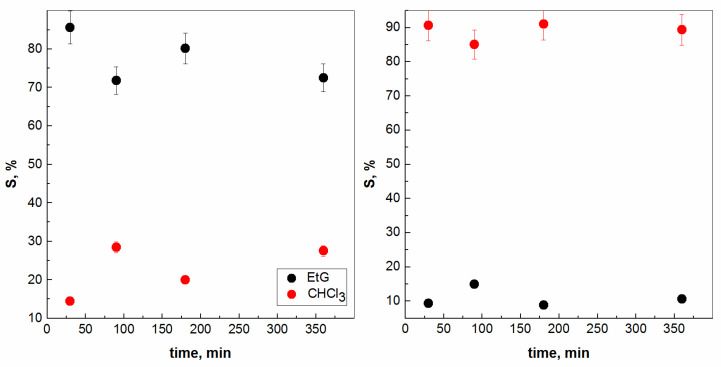
Mutual solubility between chloroform and ethylene glycol vs. contacting time (**left**: CHCl_3_ → EtG; **right**: EtG → CHCl_3_).

**Figure 4:**
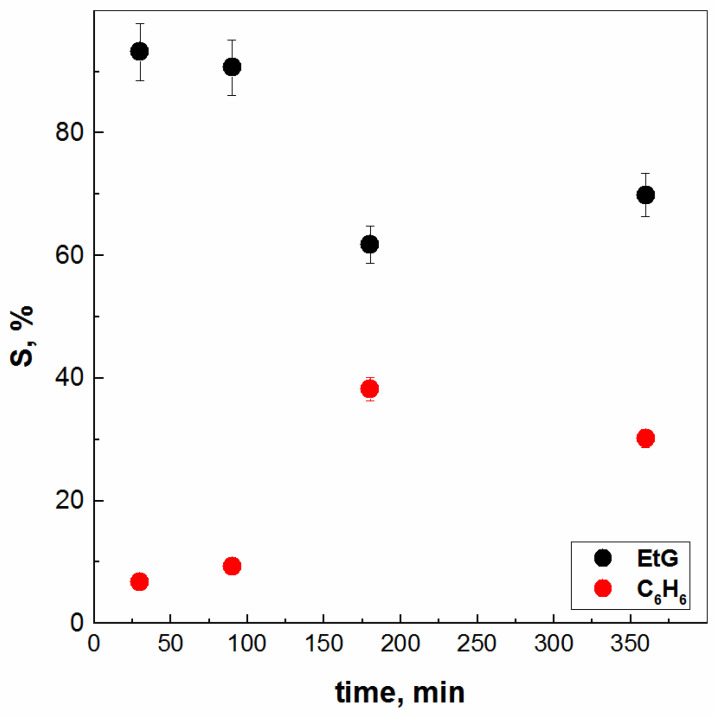
Mutual solubility between benzene and ethylene glycol vs. contacting time.

However, additional experiments were performed also considering the contact time between the two liquid phases, presented in Figure 3, exactly with chloroform. In general, the average profit value was about 11% in the direction of EtG transferring to CHCl_3_, regardless of the contact time (30 min, 90 min, 180 min, or 360 min). At the same time, it could be concluded and assumed that the maximum percentages reported for the solubility of CHCl_3_ in EtG are up to ca. 28% again, regardless of the time without the first experiment, i.e., 30 min. A similar behaviour could be expected when using benzene with an increase in the contact time between the two liquid phases (Figure 4). Experimentally, it was found that the diffusion process becomes less likely within the frame of 90 min, with a lower miscibility of C_6_H_6_ in EtG. At the same time, the migration of EtG molecules into C_6_H_6_ is negligible (~4%) or 0%. Needless to say, as might be expected, one should emphasize again that this VOC is already prohibited for use in all branches of chemical technology and will remain exotic or only for comparative purposes in science, such as the present study.

**Figure 5:**
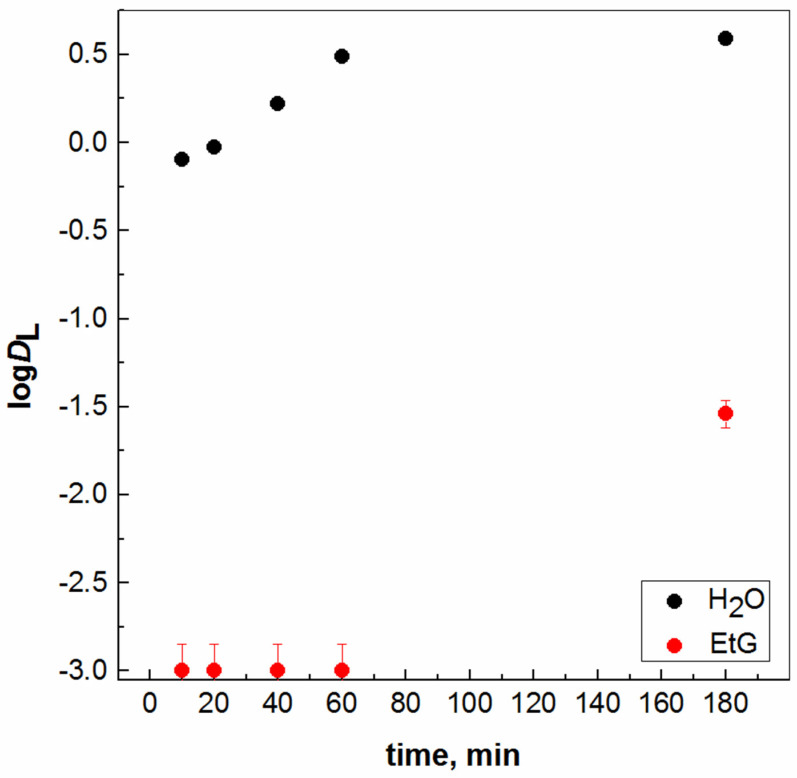
Log*D*_L_ for Gd(III) ion extracted with [HP] = 4 × 10^−2^ mol/dm^3^ from H_2_O or EtG phase (0.1 mol/dm^3^ MES) vs. time: kinetic study.

A kinetic study was also carried out for the solvent extraction of Gd(III) ion with the chelating ligand 4-benzoyl-3-methyl-1-phenyl-2-pyrazol-5-one (HP) from a more polar phase, i.e., aqueous phases or EtG (MP). Solvent extraction has been widely employed as a versatile separation method for metal ions in various fields from analytical chemistry to hydrometallurgy because of its simplicity, speed, and applicability to both trace and macro amounts of metal target species. Once the two phases are mixed adequately, ensuring excellent kinetics, they separate easily because solvent extraction is an interfacial phenomenon. Despite the great variety of solvent systems, it is possible to describe every extraction process using a simple, three-step scheme. Thus, an understanding of the physicochemical properties of (usually) the aqueous electrolyte and of organic solutions, as they determine the role of the interactions with the solute, is necessary for the successful design of solvent extraction systems. Even so, organic losses due to the extractant or diluent molecules can occur through a number of physical and chemical mechanisms during a solvent extraction circuit implementing VOCs, and thus, always present engineering challenges in ensuring the minimization of solubility losses. From the dependencies obtained, the need to increase the contact time between two liquid layers is emphasized again in order to transfer the ion from the MP phase to the other organic, less polar (LP) phase, in contrast to an aqueous medium, where 50% extraction is generally achieved in 20 min (Figure 5) [22]. As the results above belong to lab-scale experiments, it will be better to continue for the benefit of green extraction processes provided by ionic liquid compounds as well. A similar research investigation with ionic liquid compounds is underway in our laboratory.

Recently, a typical method was introduced for the intragroup separation of heavy rare earths from light ones via the successful replacement of water with the polar organic diluent ethylene glycol in the presence of LiNO_3_ up to 1 mol/dm^3^ or LiCl up till 4 mol/dm^3^, while the extracting organic phase consists of Cyanex 923 ligand dissolved in n-dodecane [5,43,44]. The achieved efficient separation was highly pronounced in comparison to water samples; per contra, it was impossible to omit the moderate toxicity of the proposed organic entity and the relative expense of the process. Since two organic immiscible phases were used for solvent extraction, being aware of this point, their mutual solubility was also estimated by the authors in a similar manner by applying ^31^P NMR and gas chromatography. The mutual solubility of ethylene glycol and n-dodecane was measured by recording the ^1^H NMR spectra of both phases after a 30 min equilibrium of equal volumes (5 mL), with no resonance line observations revealing completely immiscible liquids. Furthermore, ^31^P NMR spectra indicated no loss of the Cyanex 923 extractant in the ethylene glycol phase. However, it was found that about 24.5 g·L^−1^ of EtG was coextracted into 1 mol/dm^3^ Cyanex/n-dodecane, estimated with GC. This coextracted content decreased to 20.8 g·L^−1^ from the addition of 1 mol/dm^3^ LiNO_3_ and 10 g·L^−1^ La(III) ion to the EtG phase [43]. The solubility/coextraction of ethylene glycol diluent by 1 mol/dm^3^ Cyanex 923/(+10% *v*/*v* 1-decanol)/n-dodecane was measured to be significant: 46.3 g·L^−1^ (1-decanol was added as a phase modifier). This value decreased to 29.7 g·L^−1^ upon the addition of 2 mol/dm^3^ LiCl and was further suppressed to 23.5 g·L^−1^ when extractions were carried out from feed solutions of 50 g·L^−1^ rare earths together with 2 mol/dm^3^ LiCl. Perhaps it is important to underline that the coextraction of water and ethylene glycol is comparable [44]. In addition, Binnemans’s group investigated the extraction mechanism in detail with the two metallic ions Yb^3+^ and Nd^3+^, under the same experimental protocols, using the slope analysis method and extended X-ray adsorption fine structure (EXAFS). The established extracted species in the Cyanex 923(L)/n-dodecane phase were different and demonstrated divergent coordination: YbCl_3_·4L and Nd(NO_3_)_3_·3L·EG, where EG is an ethylene glycol molecule [43].

### 2.2. NMR Investigation

Parallel studies were also performed and the samples were analysed using ^1^H NMR spectra (see Supplementary Material, Figures S13–S18). It should be noted, again, that the samples were dissolved in acetone-d_6_ (0.05 mL in 0.5 mL of acetone) as already mentioned in the experimental part. The ^1^H NMR spectra of the different organic liquid phases were recorded. The amount of dissolved diluent was quantified by comparing the integral intensities of the peaks belonging to different molecules. The obtained results are presented in Table 1. As expected, a negligible presence of EtG was detected in the three organic diluents tested. The calculated amounts (in %) were less than those obtained with gas chromatography. What is debatable, however, is the resulting difference in solubilities of C_6_H_6_ in ethylene glycol using the two analytical methods: 3.9% using NMR vs. 38.2% using GC. These discrepant data can be explained by the underestimation of ethylene glycol content due to higher values of response factors for oxygenated compounds in respect to those for hydrocarbons [45,46]. The good thing is that for 1,2-dichloroethane, there was almost a complete match in the results if we compare the data presented in Figure 1 and those in Table 1. Therefore, we can accept them without objection. An interesting fact is that when using the NMR spectra for an estimation of the solubility of CHCl_3_ (lower layer) in the upper layer, namely ethylene glycol, the values were slightly higher than those calculated from GC, with the reported difference being approximately 5%. 

It has to be noted, as well, that the above-described experiments were performed with anhydrous ethylene glycol, as confirmed by the spectra (see Supplementary Material, Figures S14, S16, and S18). When using the wet one, its solubility in the organic diluent was analogous but the amount of the organic diluent in EtG was somehow significantly reduced by almost twice as much. The latter was most probably due to the insolubility of benzene, chloroform, and dichloroethane in water (the presence of water in EtG).

Moreover, the study was extended to include the compounds 1,2- and 1,3-propandiol as potential future substitutes for the aqueous phase. Of course, their solubility in some organic diluents was significant, with a visible volume change in both liquid phases. Therefore, this limited the study to nonpolar cyclohexane and the possibility to analyse chloroform as well, but unfortunately, only in one of the cases. The obtained results are presented in Table 2 (see Supplementary Material, Figures S19–S24). Unfortunately, the migration of CHCl_3_ into the 1,3-propandiol liquid phase is significant and should be considered in future research investigations. At the same time, C_6_H_12_ is quite suitable, and it should be noted that there is no chlorine atom in its composition. It is probably worth noting that the order of solubility of the tested organic compounds in C_6_H_12_ is EtG > 1,2-propandiol > 1,3-propandiol. This could be an interesting result, especially for future industrial applications of nonaqueous solvent extraction systems.

## 3. Materials and Methods

### 3.1. Materials

All reagents were purchased from Aldrich, Merck, and Fluka and were used without further purification. The diluents were CHCl_3_ (Merck, p.a.), C_6_H_6_ (Merck, 99.7%), C_6_H_12_ (Merck, p.a.), CCl_4_ (Fluka, p.a.), C_2_H_4_Cl_2_ (Merck, p.a.), and CH_3_(CH_2_)_4_CH_3_, i.e., n-hexane (Merck, ≥95%), ethylene glycol (Merck, 99.5%), 1,2-propanediol (Sigma-Aldrich, 99%), and 1,3-propanediol (Aldrich, 98%). The commercial product 4-benzoyl-3-methyl-1-phenyl-2-pyrazolin-5-one (HP, purity > 99%, Fluka) was used as received. The deuterated acetone was purchased from Deutero GmbH. All other commercially available analytical-grade reagents were used without any further purification.

A stock solution of gadolinium ion (3 × 10^−3^ mol/dm^3^) was prepared from Gd(NO_3_)_3_∙6H_2_O (Fluka, puriss) by dissolving and diluting with distilled water to the required volume. 65% nitric acid was used (Merck, p.a.) to adjust the pH of the aqueous solutions or nonaqueous phase added to 0.1 mol/dm^3^ 2-morpholinoethanesulfonic acid (MES) buffer (Alfa Aesar, 98%). 

### 3.2. Analytical Methodology

For binary systems containing ethylene glycol (or 1,2-/1,3-propanediol) and organic diluents, the mutual solubility between them was determined at room temperature (22 ± 2 °C). For the investigated systems, 2.5 mL of the two organic liquids was used and gently stirred (1500 rpm) for 3 h (or the specified different time during the kinetic investigation). After mixing, the samples were allowed to rest for at least 30 min to achieve separation. Following the centrifugation (Micro Star 12) of each liquid phase, an aliquot was taken up for gas chromatography. The gas chromatograph “KONIK-TECH”–Spain model MODEL 4000 B was used, equipped with a flame ionization detector and N-5 204 capillary column (Nordion Instruments Oy Ltd.) that was 25 m long with an internal diameter of 0.32 mm and a stationary phase thickness of 0.25 μm. The injector temperature was 330 °C. High-purity helium was used as the carrier gas at a gas flow rate of 1 mL/min. The initial column temperature was 25 °C, which was held for 5 min, followed by heating at 5 °C/min to 150 °C, holding for 5 min, and heating at 10 °C/min to 280 °C. The content of organic compounds in each sample in % was determined by a comparison of the peak area to that of a series of standards measured as well (see Supplementary Material, Figures S1–S12). The mutual solubilities of ethylene glycol/benzene, ethylene glycol/chloroform, ethylene glycol/dichloroethane, 1,3-propandiol/chloroform, 1,3-propandiol/cyclohexane, and 1,2-propandiol/cyclohexane biphasic systems were also determined using proton NMR spectra. The samples were recorded on a Bruker Avance NEO 400 spectrometer (Rheinstetten, Germany) in acetone-d_6_ solutions: 0.05 mL of the sample in 0.5 mL of acetone. The amounts of dissolved diluents were quantified by comparing the integral intensities of the peaks belonging to different molecules.

### 3.3. Kinetic Solvent Extraction Study

The extraction experiments were carried out at room temperature by mixing the two immiscible phases in a 1:1 *v*/*v* ratio (1.5 mL) for various investigated times (1500 rpm). After the separation of the liquid phases, the Gd(III) ion concentration in the aqueous or more polar organic phase (MP) was determined by using inductively coupled plasma–optical emission (ICP-OES) spectroscopy (“Prodigy” High dispersion ICP-OES, Teledyne Leeman Labs, USA). The concentration of the metal ion in the organic phase (the less polar phase) was obtained using a material balance. The extractant solutions in an organic phase were prepared from precisely weighted samples. The acidity of the aqueous phase at equilibrium was measured with a pH meter (pH 211 HANNA, USA) with an accuracy of 0.01 pH unit. The initial Gd(III) ion concentration was 6 × 10^−4^ mol/dm^3^ in all experiments. 

The distribution ratio (*D*) at equilibrium was calculated as:

(1) $$D=\frac{{\left[M^{n+}\right]}_{aq,in}-{\left[M^{n+}\right]}_{aq,f}}{{\left[M^{n+}\right]}_{aq,f}}\times \frac{V_{aq}}{V_{o}}$$

where [*M^n^*^+^]*_aq_,_in_* is the concentration of *M^n^*^+^ ion in the aqueous phase (MP phase) before the liquid–liquid extraction tests, and [*M^n^*^+^]*_aq_,_f_* is the concentration of the same metal ion in the aqueous phase after extraction. In general, *V_aq_* and *V_o_* are the volumes of the aqueous and organic phase as well as the volumes of the two immiscible phases used to perform the experiments, herein 1:1 *v*/*v*. For instance, duplicate experiments showed that the reproducibility of *D* measurements was generally within 95%.

## 4. Conclusions

The mutual solubility between the two liquid phases, aqueous−organic or nonaqueous(organic)–organic, during solvent extraction processes is a serious problem and should be assessed prior to their applications. Although, a priori, the two liquid phases are immiscible, the dissolution process is not entirely eliminated. Thus, this study was performed aiming to determine the quantity of both organic (immiscible) liquid compounds transferred in the upper or lower phase of biphasic nonaqueous systems based on ethylene glycol as well as 1,2-/1,3-propandiol. It was highlighted, by means of gas chromatography and ^1^H NMR spectroscopy in the case of the system incorporating CHCl_3_, that serious amount of this VOC passes into the other liquid phase. The investigated diffusion process becomes less likely with other tested traditional organic diluents. The obtained results clearly showed that the solubility of organic diluents in EtG decreased in the following order: C_6_H_6_ > CHCl_3_ > C_2_H_4_Cl_2_ > n-hexane > C_6_H_12_ > CCl_4._ The influence of the chemical nature and physicochemical characteristics of typical organic diluents on the dissolution process was discussed. In fact, the migration of C_6_H_6_ into the more polar phase is impressive from a scientific point of view, but without consequences, because this particular diluent is prohibited from use, i.e., it is very harmful and highly toxic, especially in industrial conditions. Therefore, based on scientific results and taking into account all environmental aspects, nonpolar and chlorine-free diluents like C_6_H_12_ or n-hexane could eventually be combined as a medium in nonaqueous solvent extraction systems for various metals. Somehow, this kind of effect might be further exploited to directly improve the diffusional motion of solute molecules, and also in solvent extraction chemistry. Work addressing these opportunities is underway in our laboratory. 

## Figures and Tables

**Table 1:** Solubility data based on ^1^H NMR and GC (in parenthesis) analyses.

Sample	Ethylene Glycol		Organic Diluent	
EtG ↔ C_6_H_6_	96.1% (61.8%)	3.9% (38.2%)	99.9% (96.2%)	0.1% (3.8%)
EtG ↔ CHCl_3_	75.5% (80.1%)	24.5% (17.1%)	98.4% (88.2%)	1.6% (8.9%)
EtG ↔ C_2_H_4_Cl_2_	90.1% (89.1%)	9.9% (10.9%)	99.5% (97.1%)	0.5% (2.4%)

**Table 2:** Solubility data based on ^1^H NMR analysis.

Sample	1,2-/1,3-Propandiol		Organic Diluent	
1,2-propandiol ↔ C_6_H_12_	97.2%	2.8%	100%	0%
1,3-propandiol ↔ C_6_H_12_	98.8%	1.2%	100%	0%
1,3-propandiol ↔ CHCl_3_	72.3%	27.7%	98.8%	1.2%

## Data Availability

Not applicable.

## Supplementary Materials

The following supporting information can be downloaded at: https://www.mdpi.com/article/10.3390/molecules28135121/s1, Figure S1. C_6_H_6_ after mixing with EtG (3 h). Figure S2. EtG after mixing with C_6_H_6_ (3 h). Figure S3. Hexane after mixing with EtG (3 h). Figure S4. EtG after mixing with n-hexane (3 h). Figure S5. C_2_H_4_Cl_2_ after mixing with EtG (3 h). Figure S6. EtG after mixing with C_2_H_4_Cl_2_ (3 h). Figure S7. CCl_4_ after mixing with EtG (3 h). Figure S8. EtG after mixing with CCl_4_ (3 h). Figure S9. C_6_H_12_ after mixing with EtG (3 h). Figure S10. EtG after mixing with C_6_H_12_ (3 h). Figure S11. CHCl_3_ after mixing with EtG (3 h). Figure S12. EtG after mixing with CHCl_3_ (3 h). Figure S13. ^1^H NMR spectrum of benzene layer after mixing with EtG in acetone-d_6_. Figure S14. ^1^H NMR spectrum of EtG layer after mixing with benzene in acetone-d_6_. Figure S15. ^1^H NMR spectrum of chloroform layer after mixing with EtG in acetone-d_6_. Figure S16. ^1^H NMR spectrum of EtG layer after mixing with chloroform in acetone-d_6_. Figure S17. ^1^H NMR spectrum of dichloroethane layer after mixing with EtG in acetone-d_6_. Figure S18. ^1^H NMR spectrum of EtG layer after mixing with dichloroethane in acetone-d_6_. Figure S19. ^1^H NMR spectrum of CHCl_3_ layer after mixing with 1,3-propanediol in acetone-d_6_. Figure S20. ^1^H NMR spectrum of 1,3-propanediol layer after mixing with CHCl_3_ in acetone-d_6_. Figure S21. ^1^H NMR spectrum of cyclohexane layer after mixing with 1,2-propanediol in acetone-d_6_. Figure S22. ^1^H NMR spectrum of 1,2-propanediol layer after mixing with cyclohexane in acetone-d_6_. Figure S23. ^1^H NMR spectrum of 1,3-propanediol layer after mixing with cyclohexane in acetone-d_6_. Figure S24. ^1^H NMR spectrum of cyclohexane layer after mixing with 1,3-propanediol in acetone-d_6_.

## Sample Availability

Samples of the compounds are not available from the authors.
