# Supplementary material for: Mutual Solubilities between Ethylene Glycol and Organic Diluents: Gas Chromatography and NMR
Source: Molecules. 2023 Jun 29;28(13):5121. doi: 10.3390/molecules28135121 (PMC10343684; doi:10.3390/molecules28135121)
Supplement: Supplementary file 1 [file molecules-28-05121-s001.zip › molecules-2470912-supplementary.pdf]

SUPPLEMANTARY MATERIAL

Mutual solubilities between ethylene glycol and organic diluents: Gas chromatography and NMR

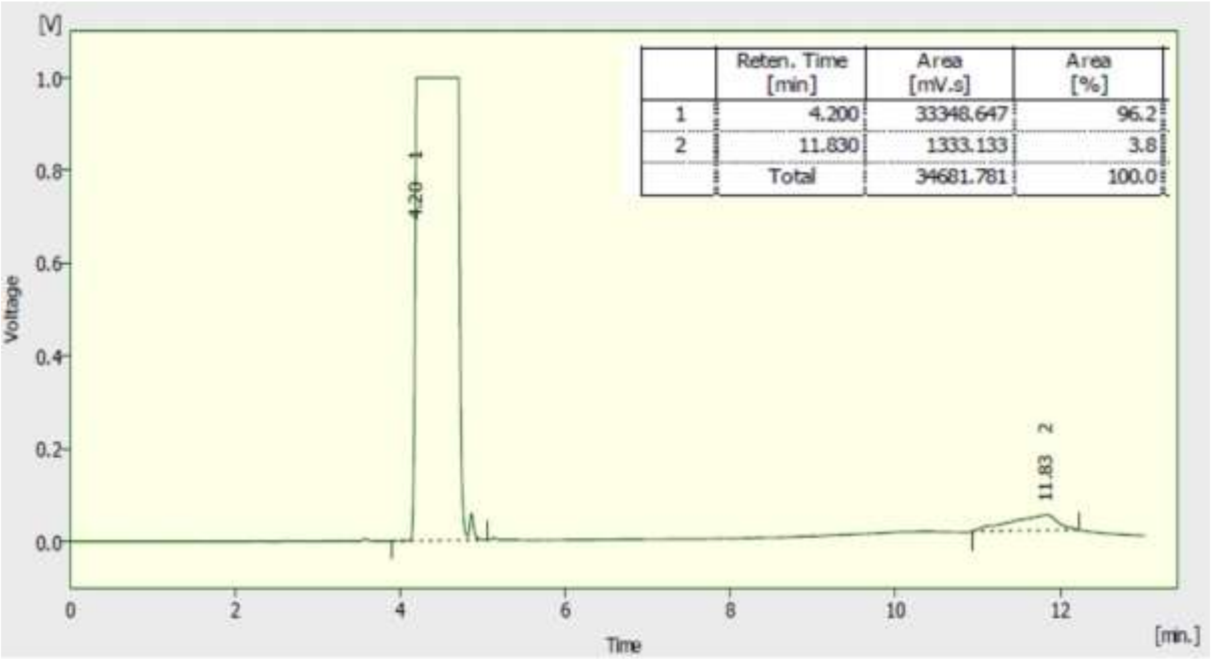

Figure S1. C_6H_6 after mixing with EtG (3h).

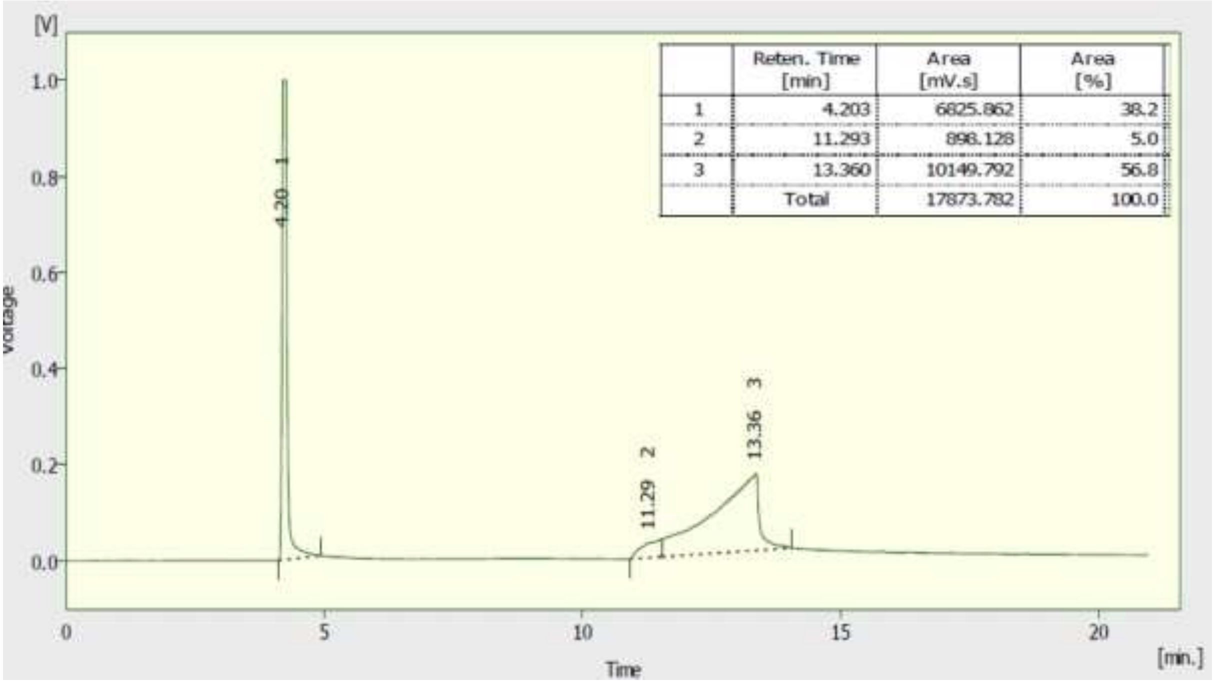

Figure S2. EtG after mixing with C_6H_6 (3h).

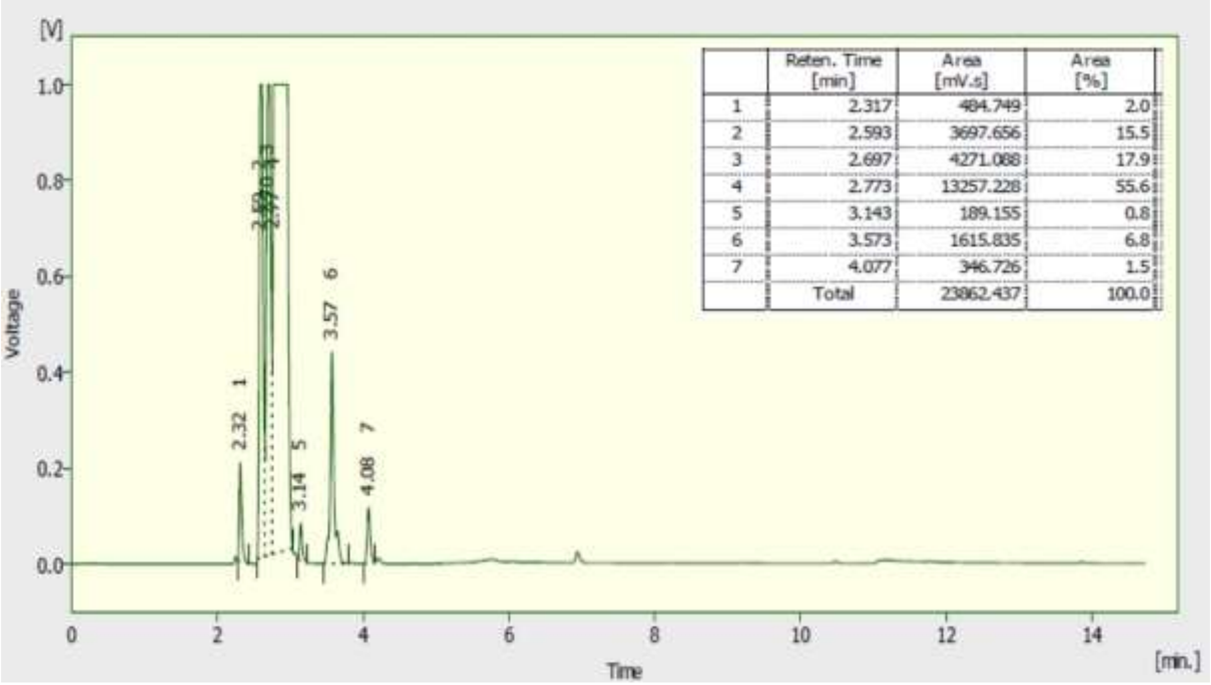

Figure S3. Hexane after mixing with EtG (3h).

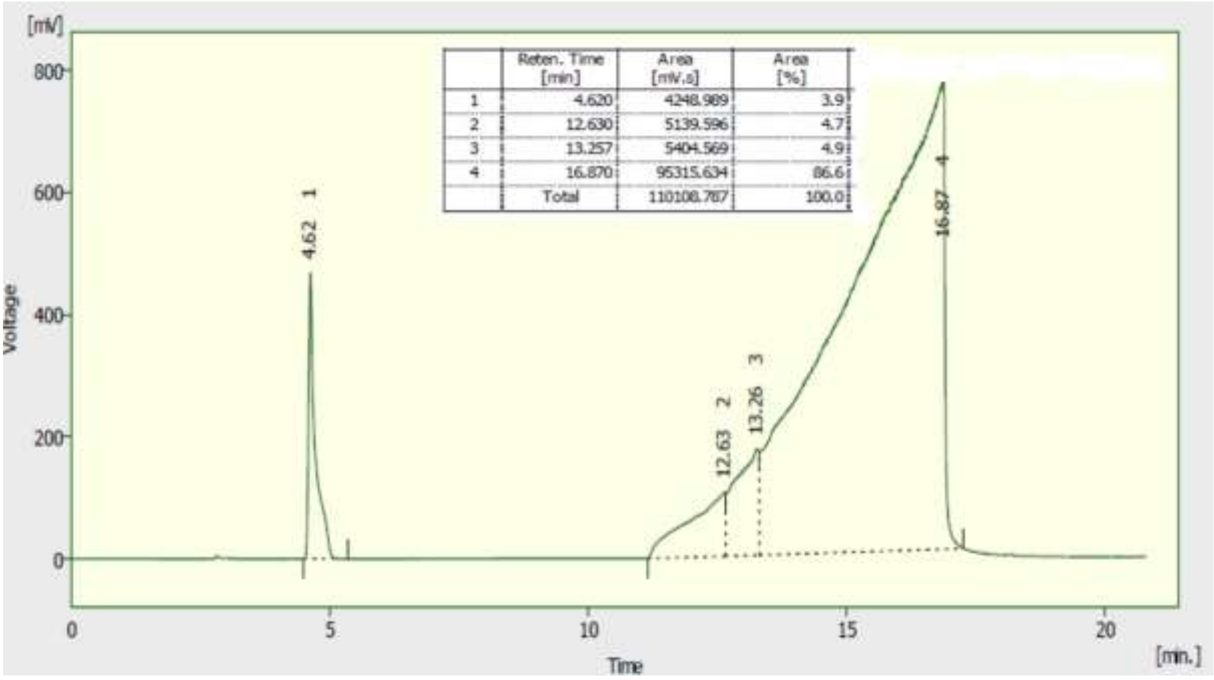

Figure S4. EtG after mixing with n-hexane (3h).

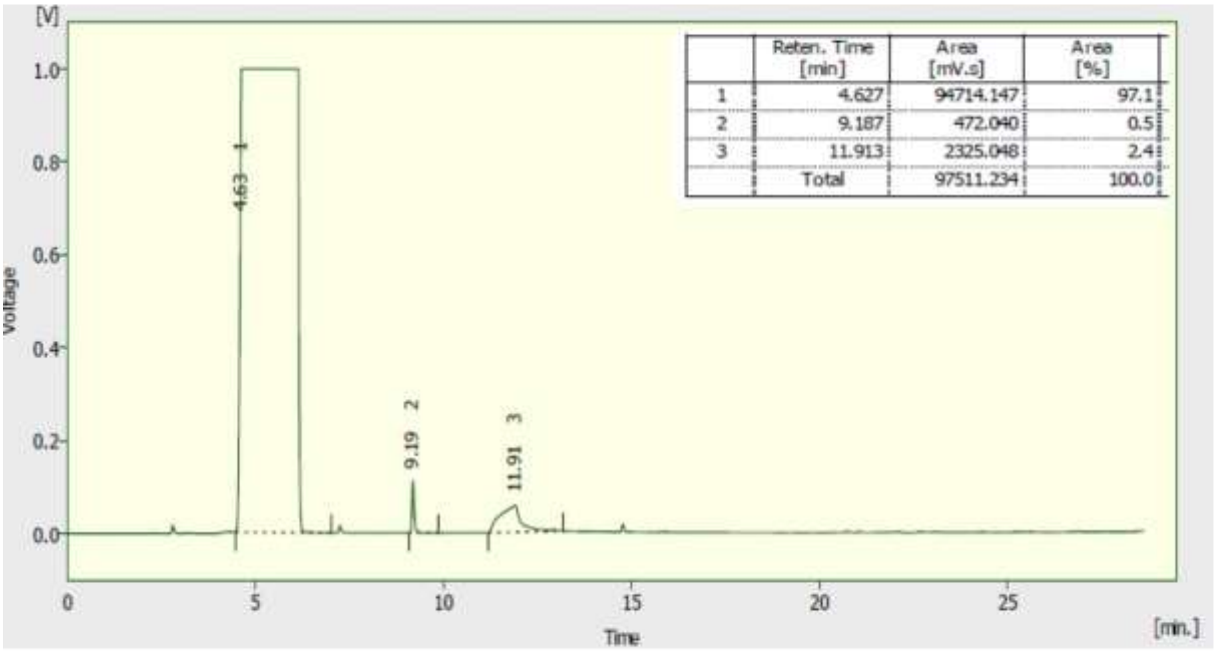

Figure S5. $C_2H_4Cl_2$ after mixing with EtG (3h).

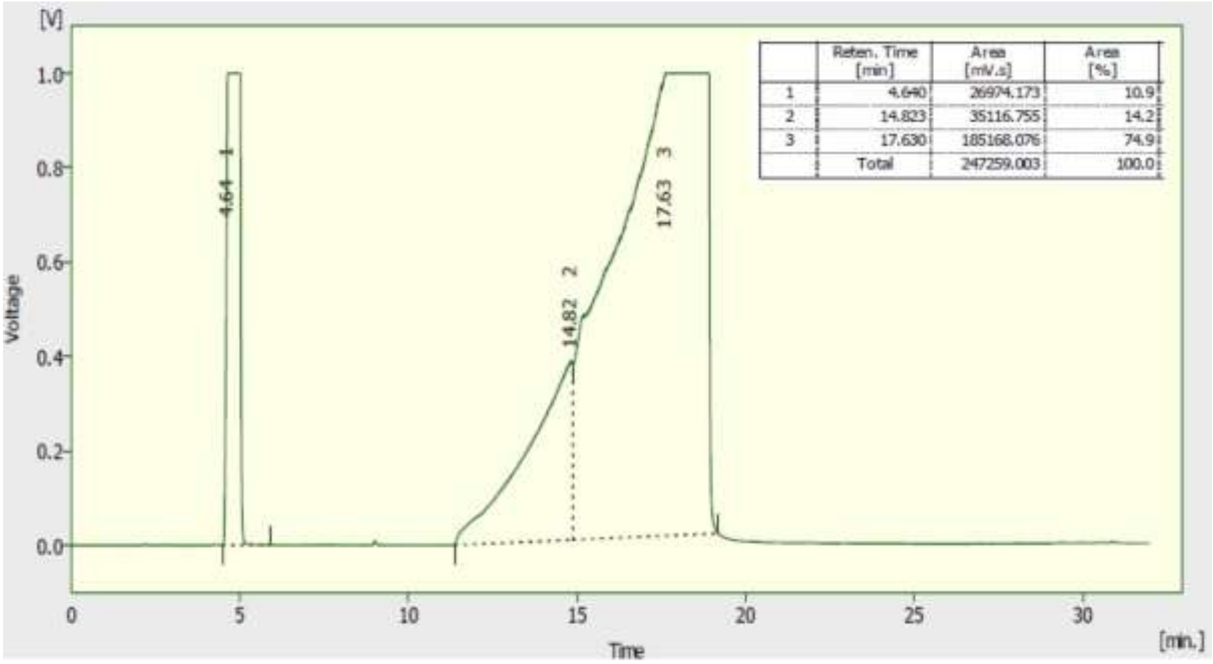

Figure S6. EtG after mixing with $C_2H_4Cl_2$ (3h).

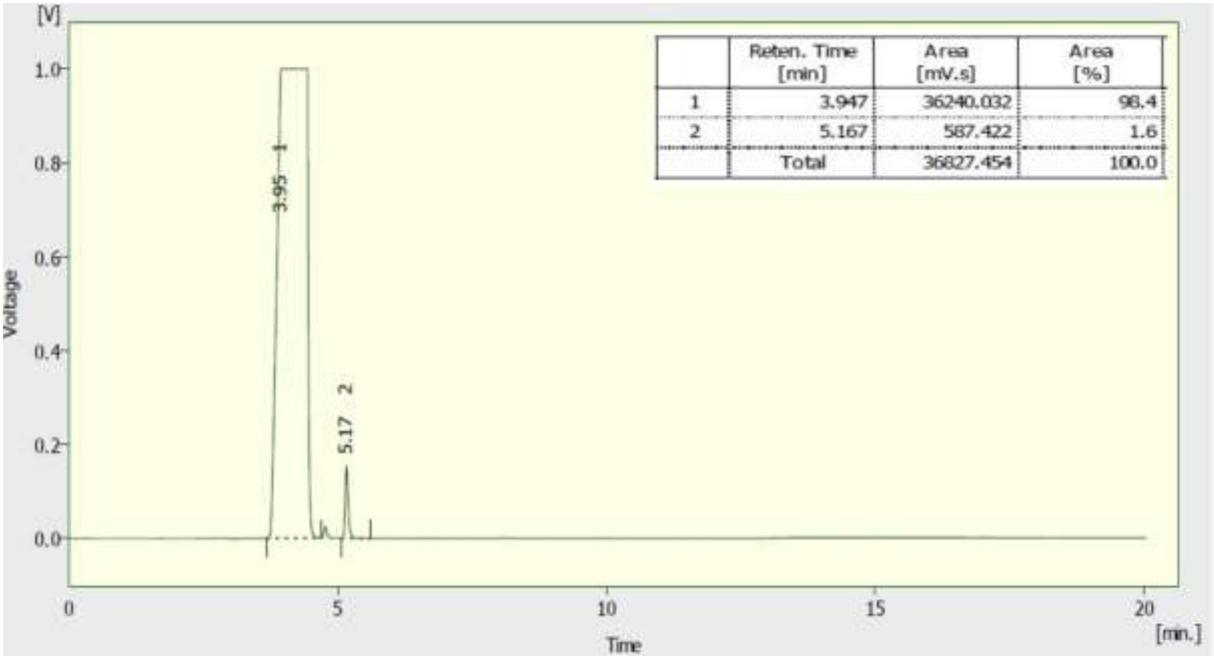

Figure S7. CCl_4 after mixing with EtG (3h).

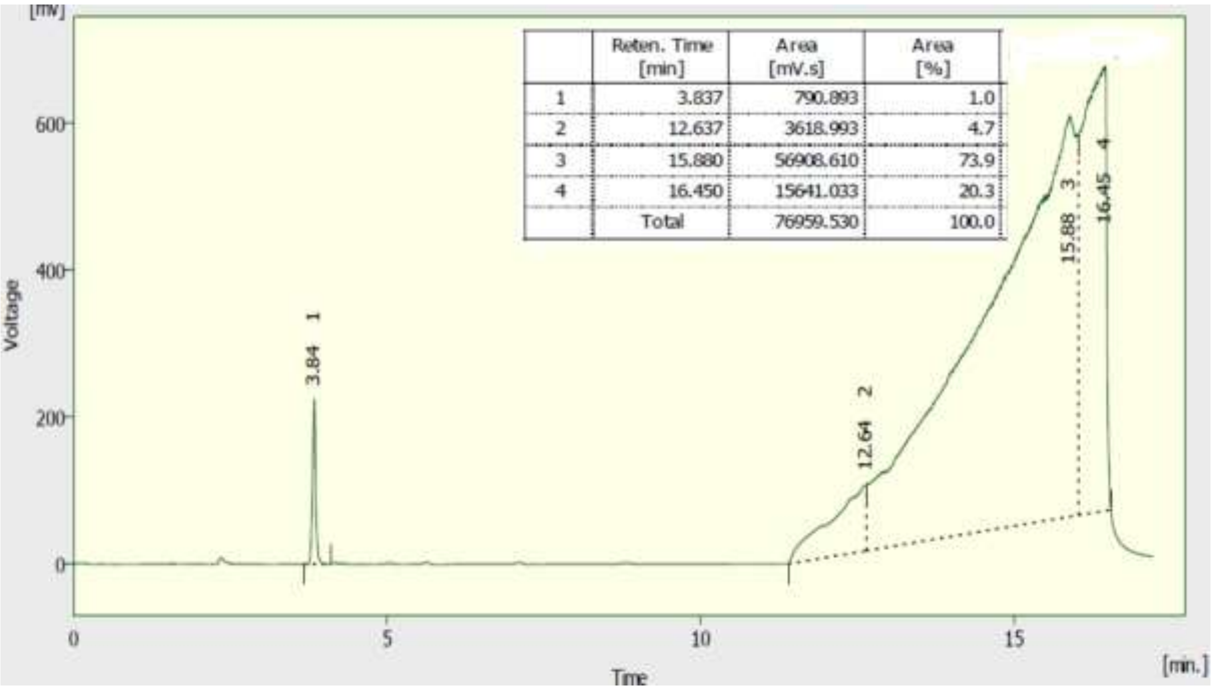

Figure S8. EtG after mixing with CCl_4 (3h).

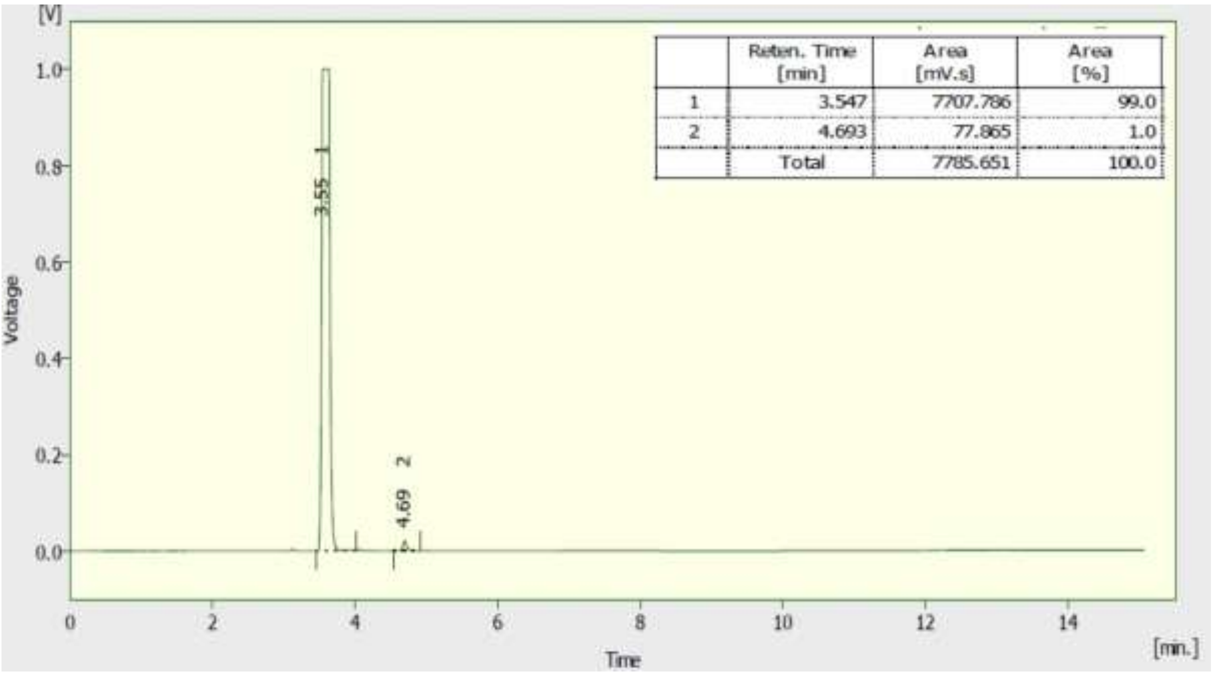

Figure S9. C_6H_{12} after mixing with EtG (3h).

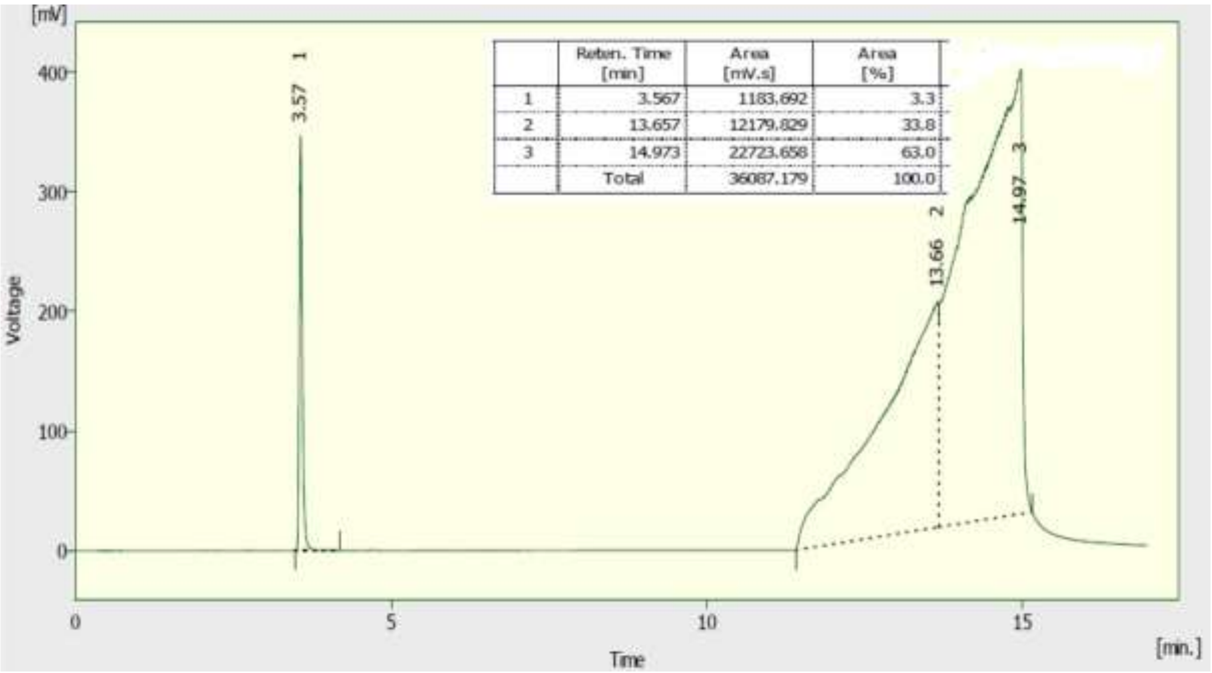

Figure S10. EtG after mixing with C_6H_{12} (3h).

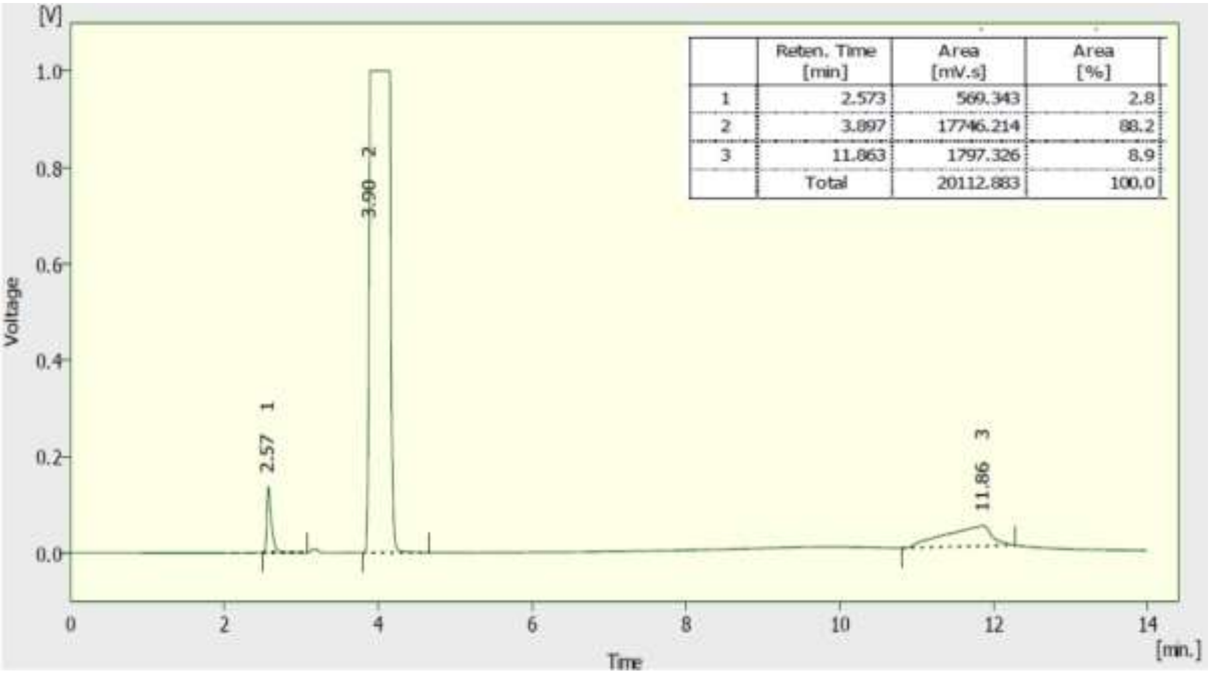

Figure S11. $CHCl_3$ after mixing with EtG (3h).

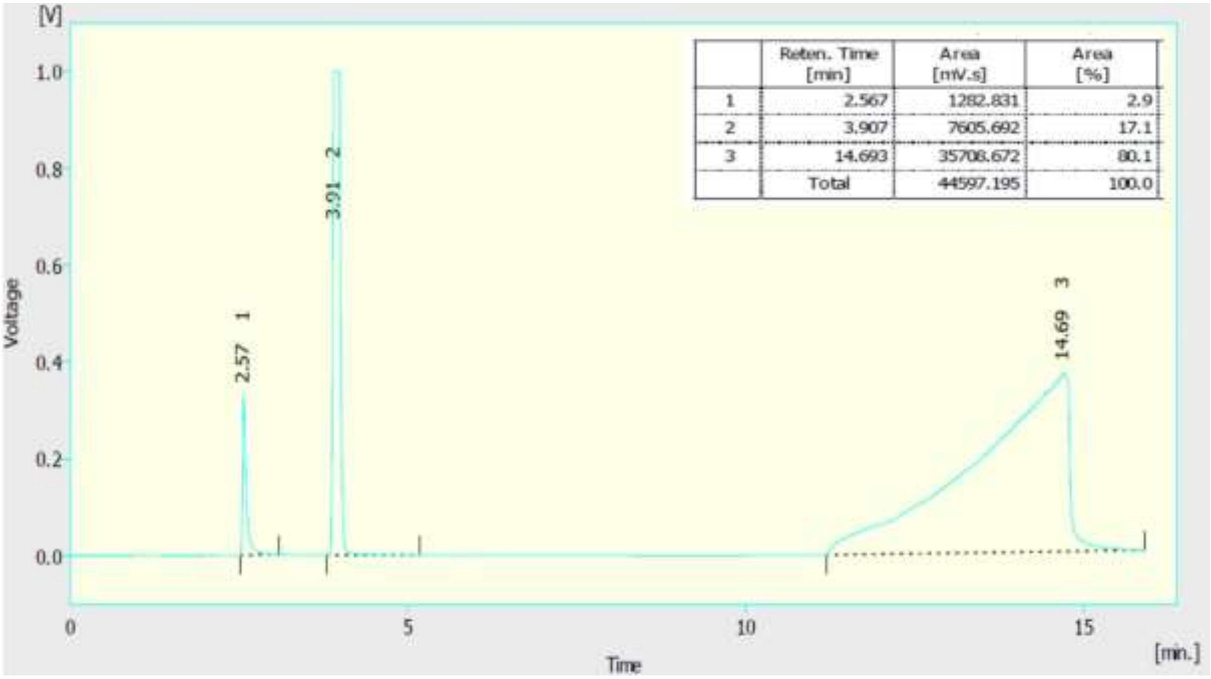

	Reten. Time [min]	Area [mV.s]	Area [%]
1	2.567	1282.831	2.9
2	3.907	7605.692	17.1
3	14.693	35708.672	80.1
	Total	44597.195	100.0

Figure S12. EtG after mixing with $CHCl_3$ (3h).

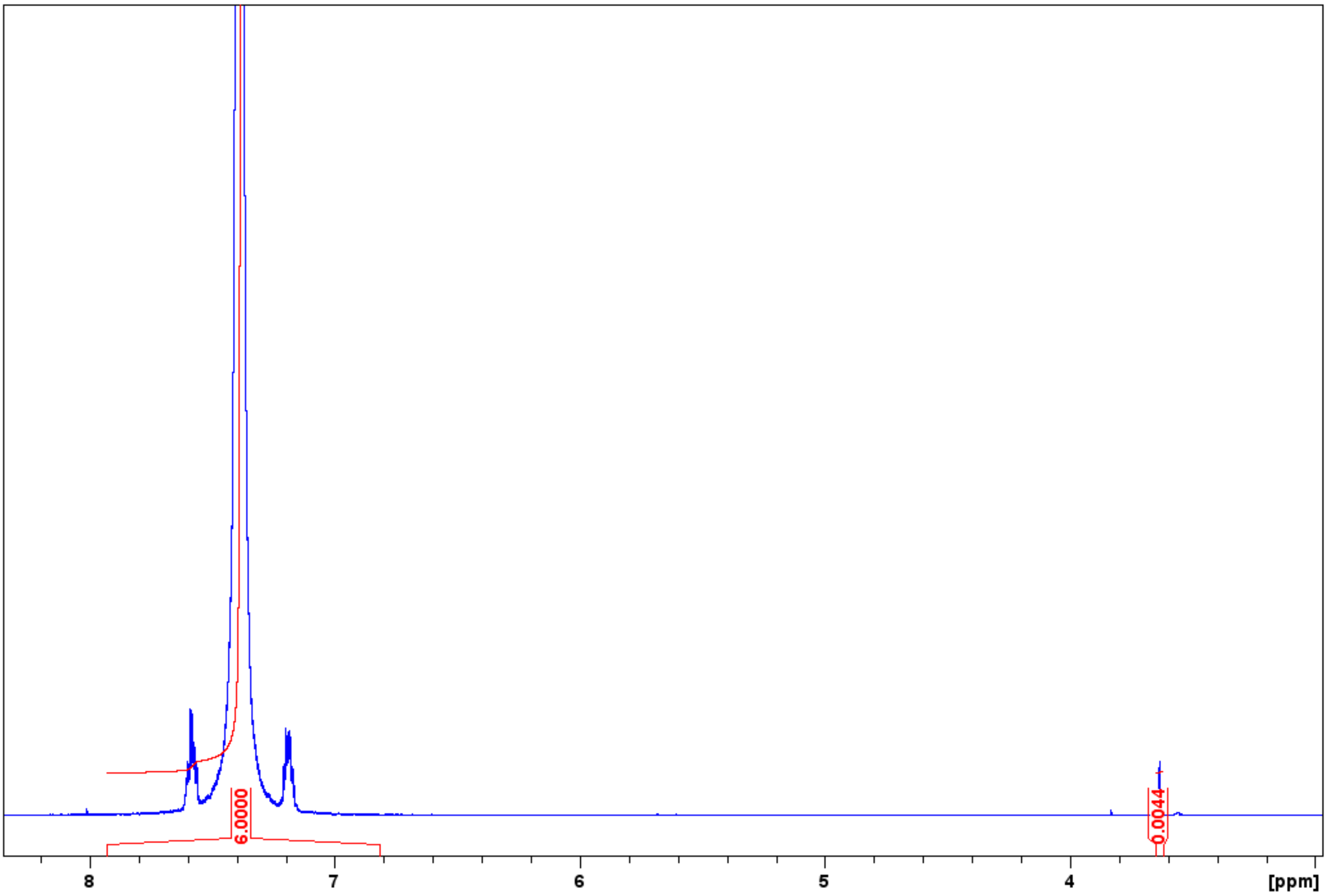

Figure S13. ^{1}H NMR spectrum of benzene layer after mixing with EtG in acetone-d_6.

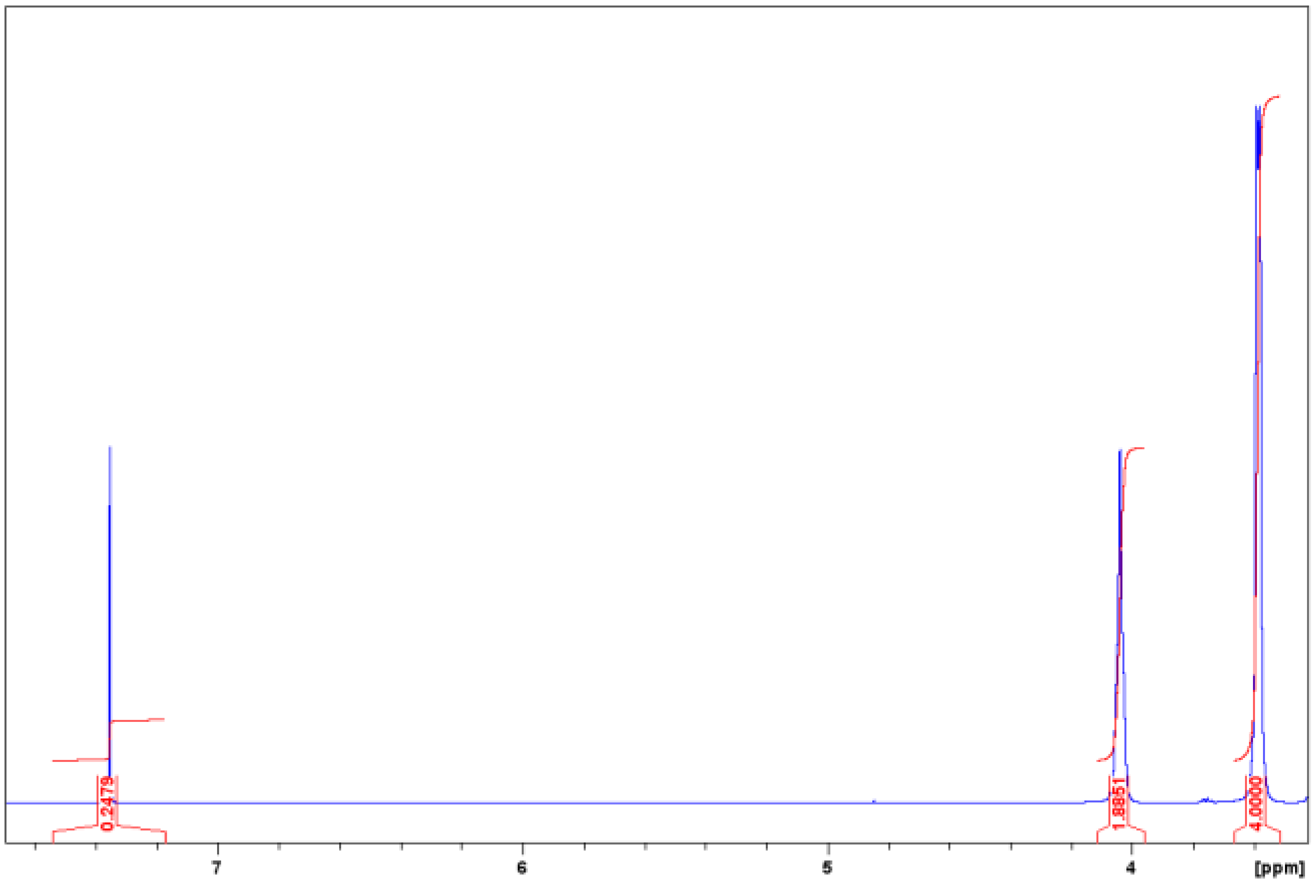

Figure S14. ^{1}H NMR spectrum of EtG layer after mixing with benzene in acetone-d_6.

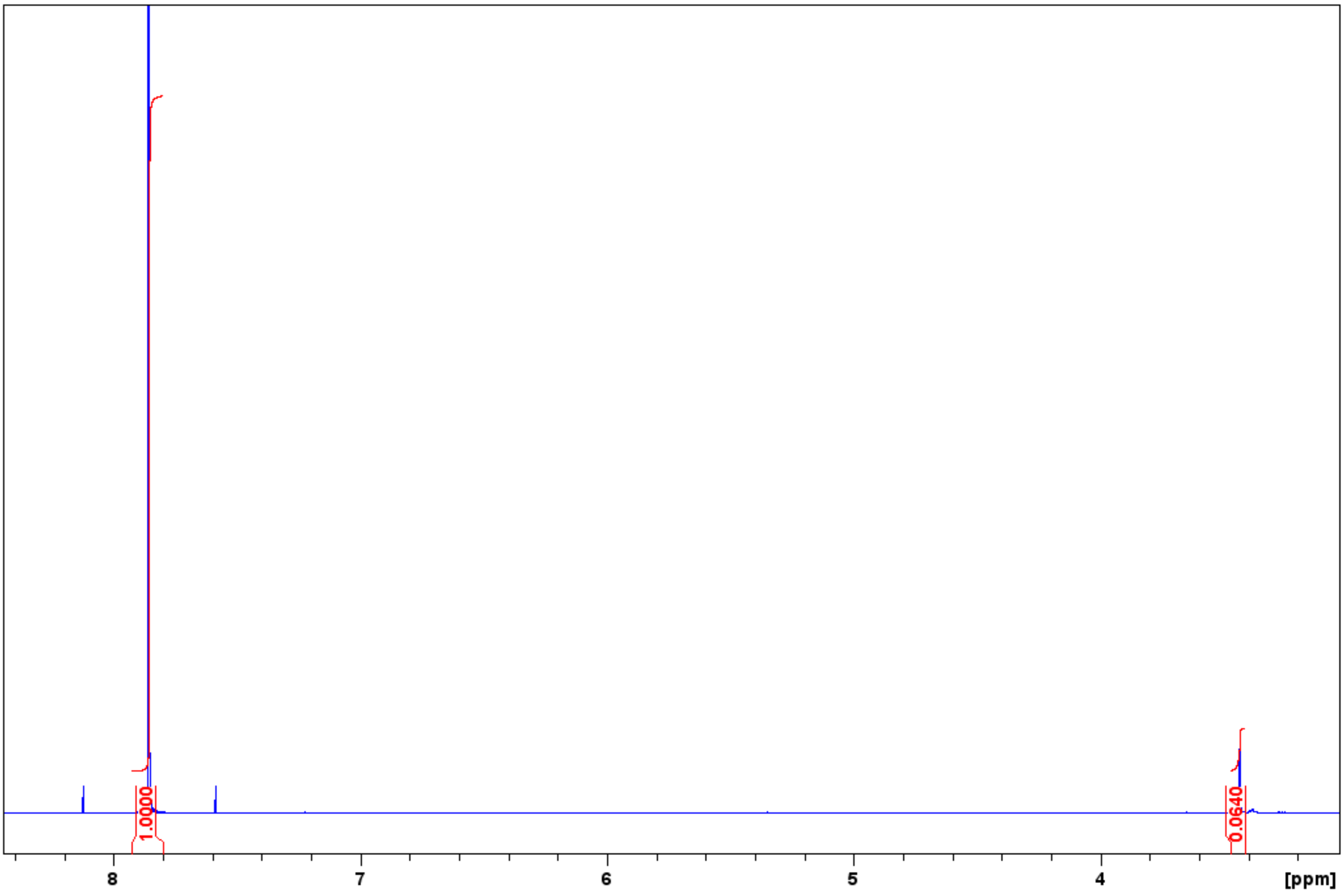

Figure S15. ^{1}H NMR spectrum of chloroform layer after mixing with EtG in acetone-d_6.

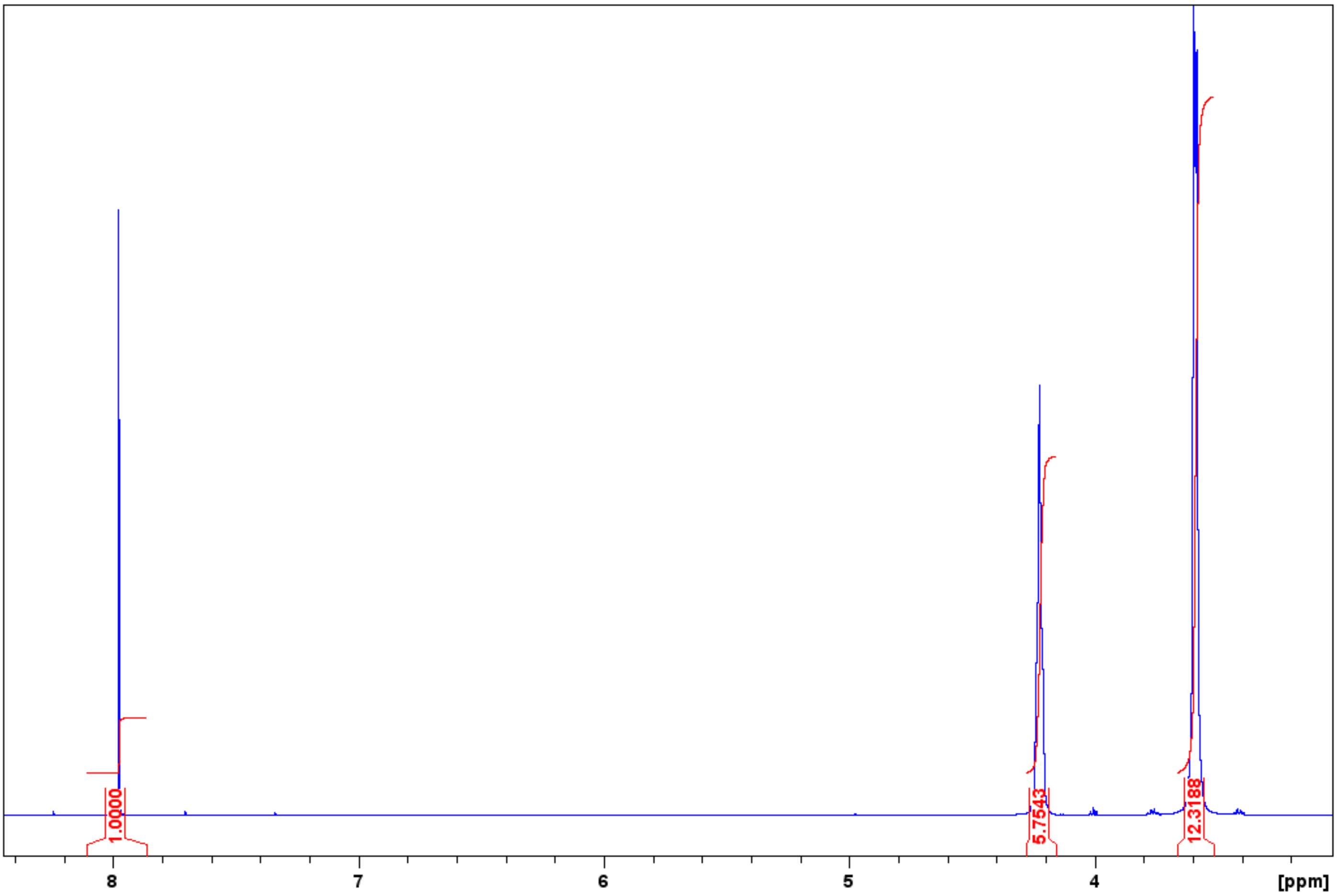

Figure S16. ^{1}H NMR spectrum of EtG layer after mixing with chloroform in acetone-d_6.

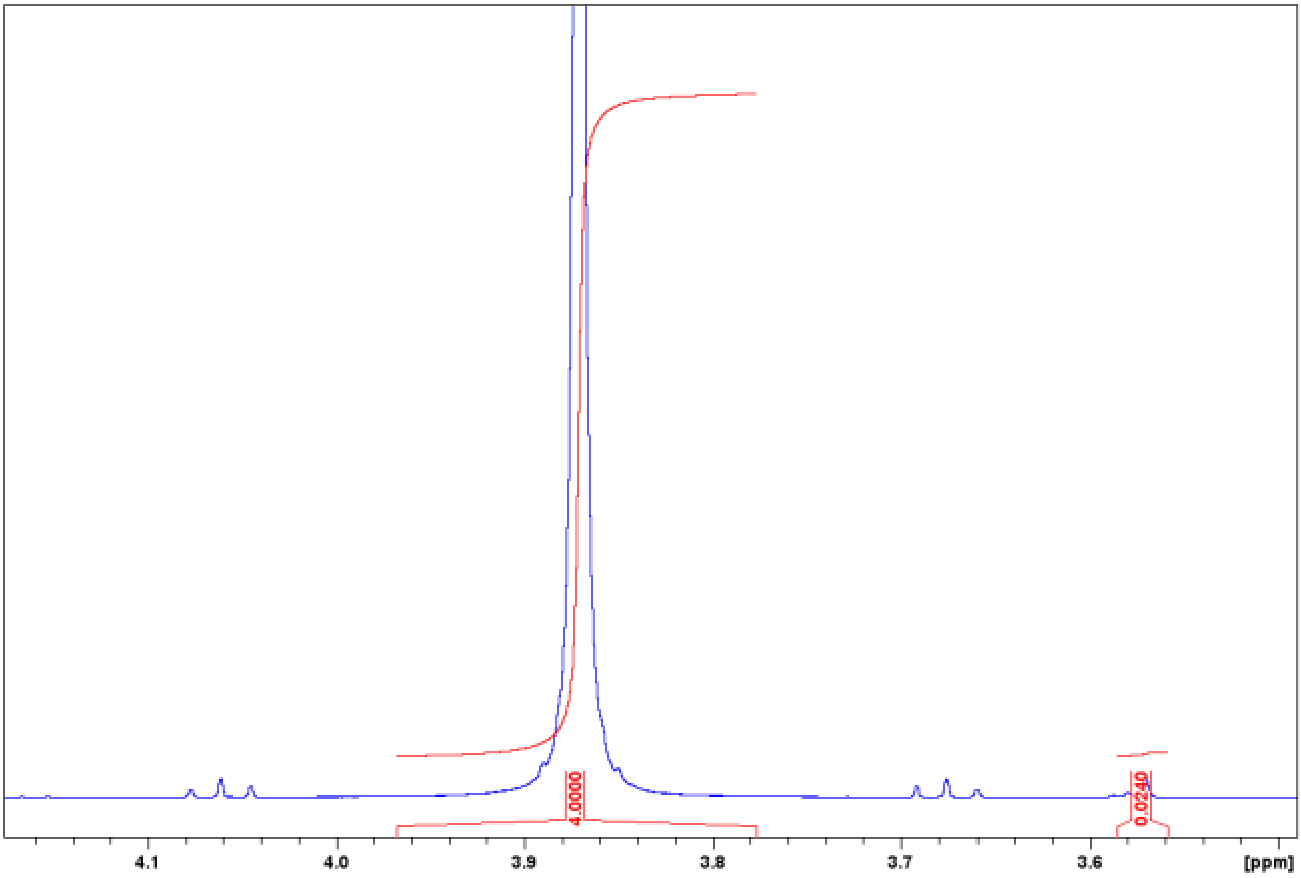

Figure S17. ^{1}H NMR spectrum of dichloroethane layer after mixing with EtG in acetone-d_6.

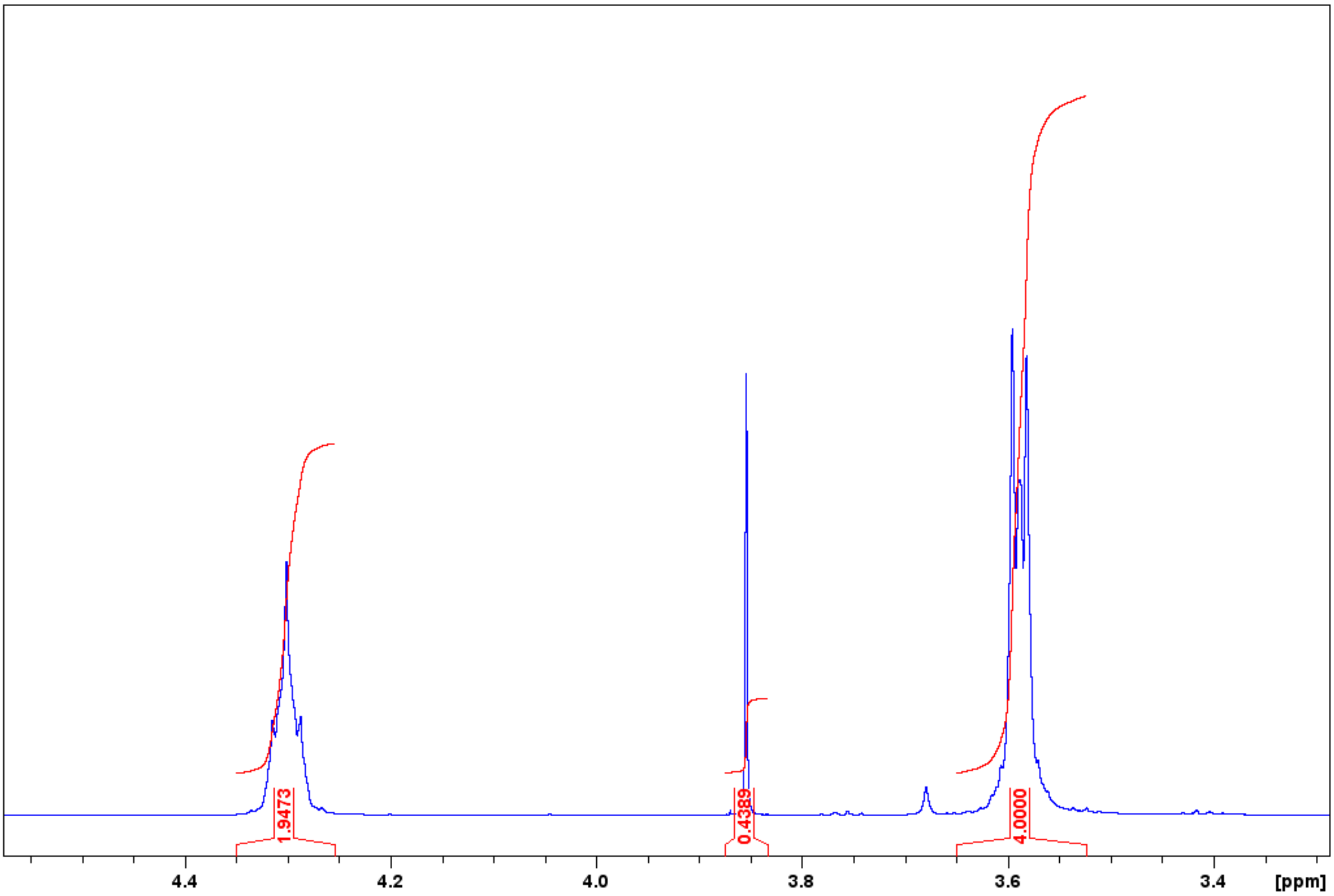

Figure S18. ^{1}H NMR spectrum of EtG layer after mixing with dichloroethane in acetone-d_6.

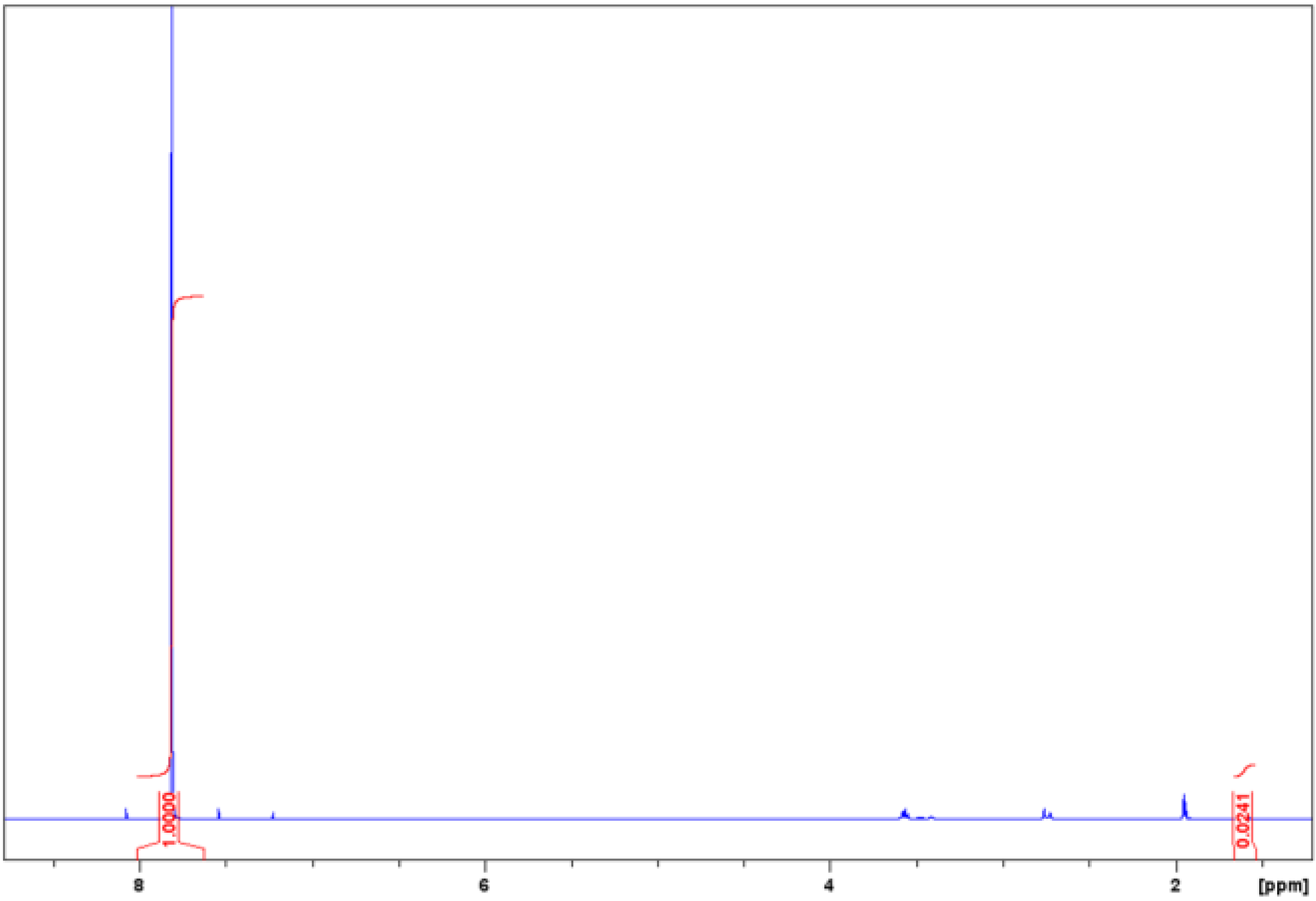

Figure S19. ^{1}H NMR spectrum of $CHCl_3$ layer after mixing with 1,3-propanediol in acetone-d_6.

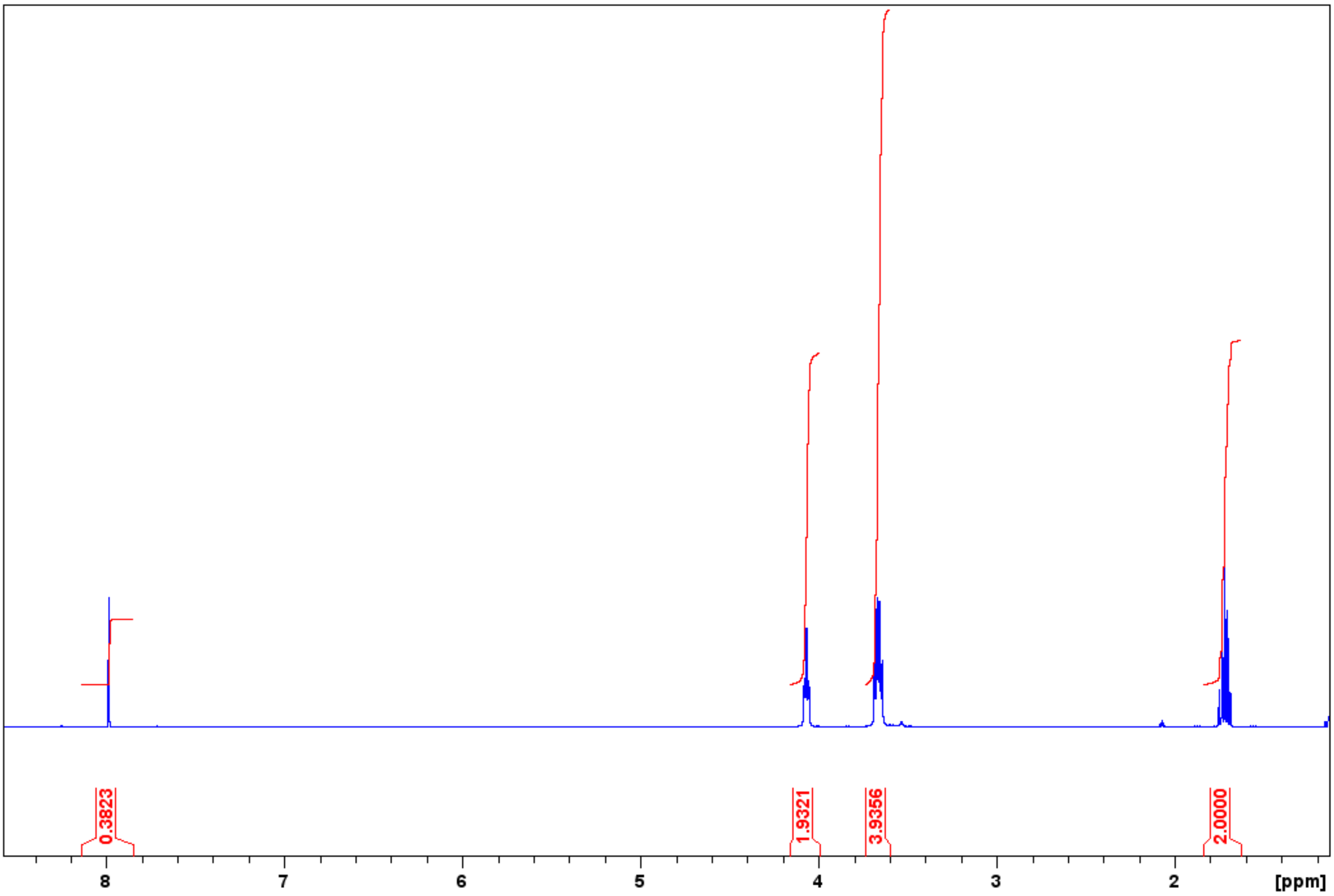

Figure S20. ^{1}H NMR spectrum of 1,3-propanediol layer after mixing with $CHCl_3$ in acetone-d_6.

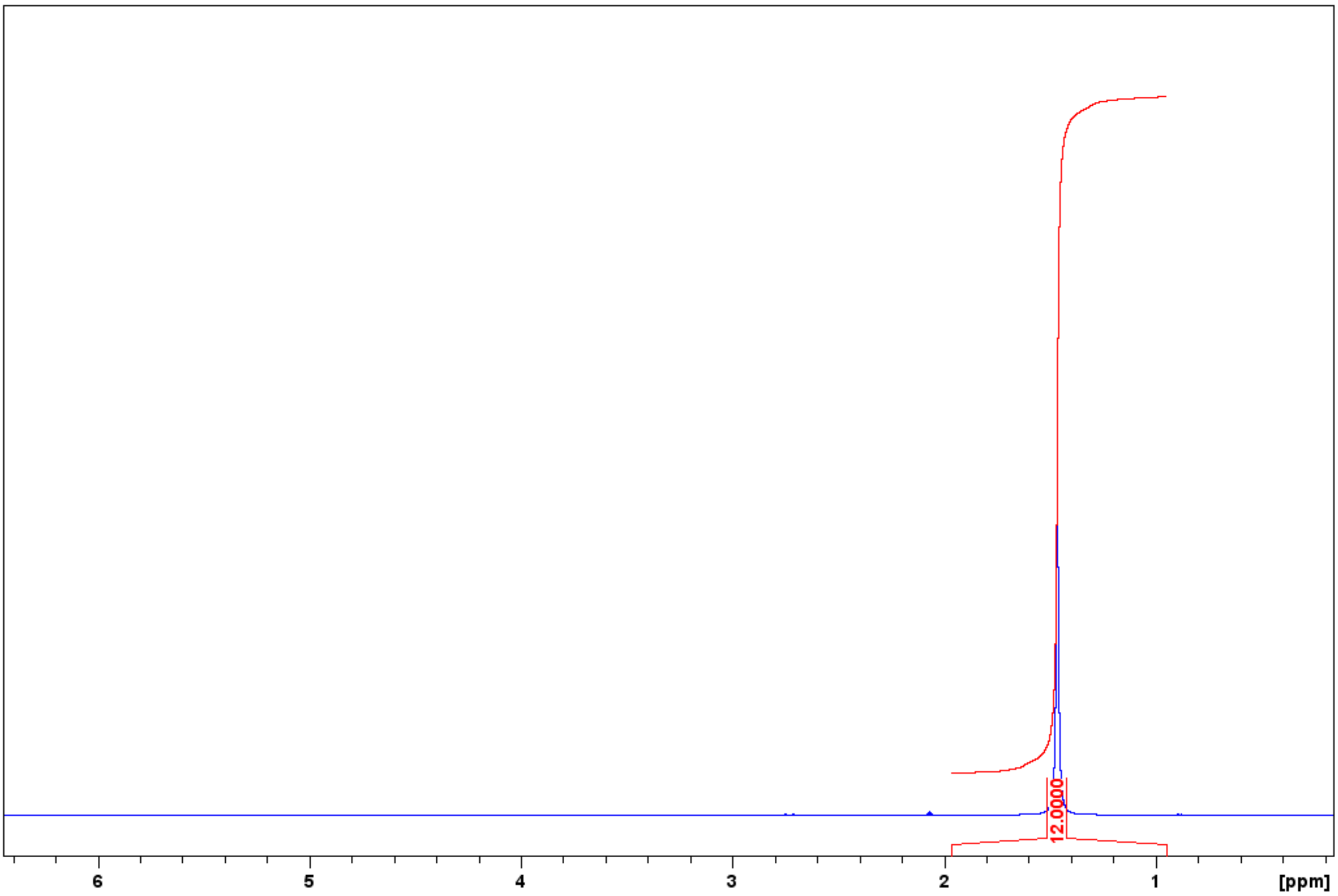

Figure S21. ^{1}H NMR spectrum of cyclohexane layer after mixing with 1,2-propanediol in acetone-d_6.

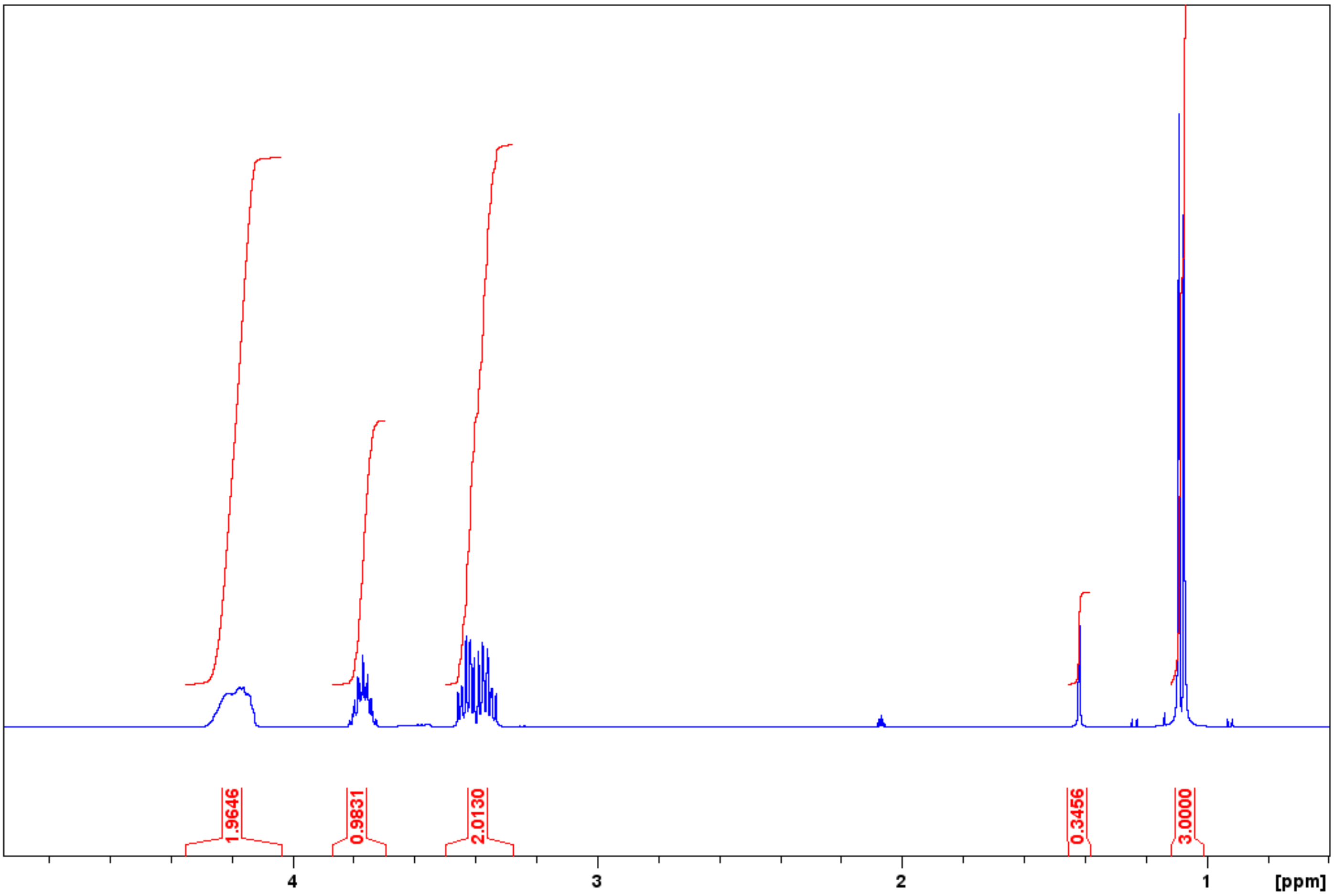

Figure S22. ^{1}H NMR spectrum of 1,2-propanediol layer after mixing with cyclohexane in acetone-d_6.

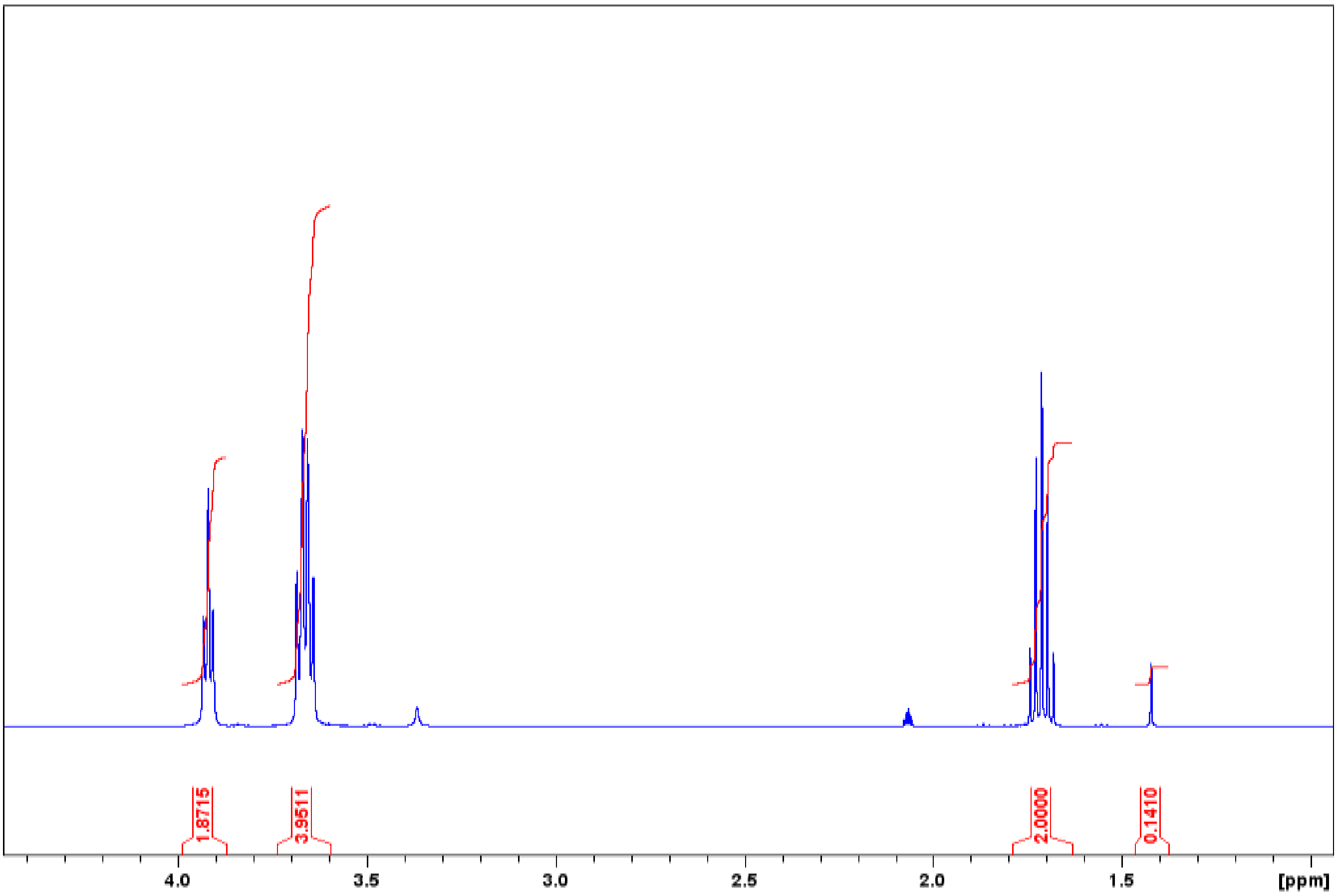

Figure S23. ^{1}H NMR spectrum of 1,3-propanediol layer after mixing with cyclohexane in acetone-d_6.

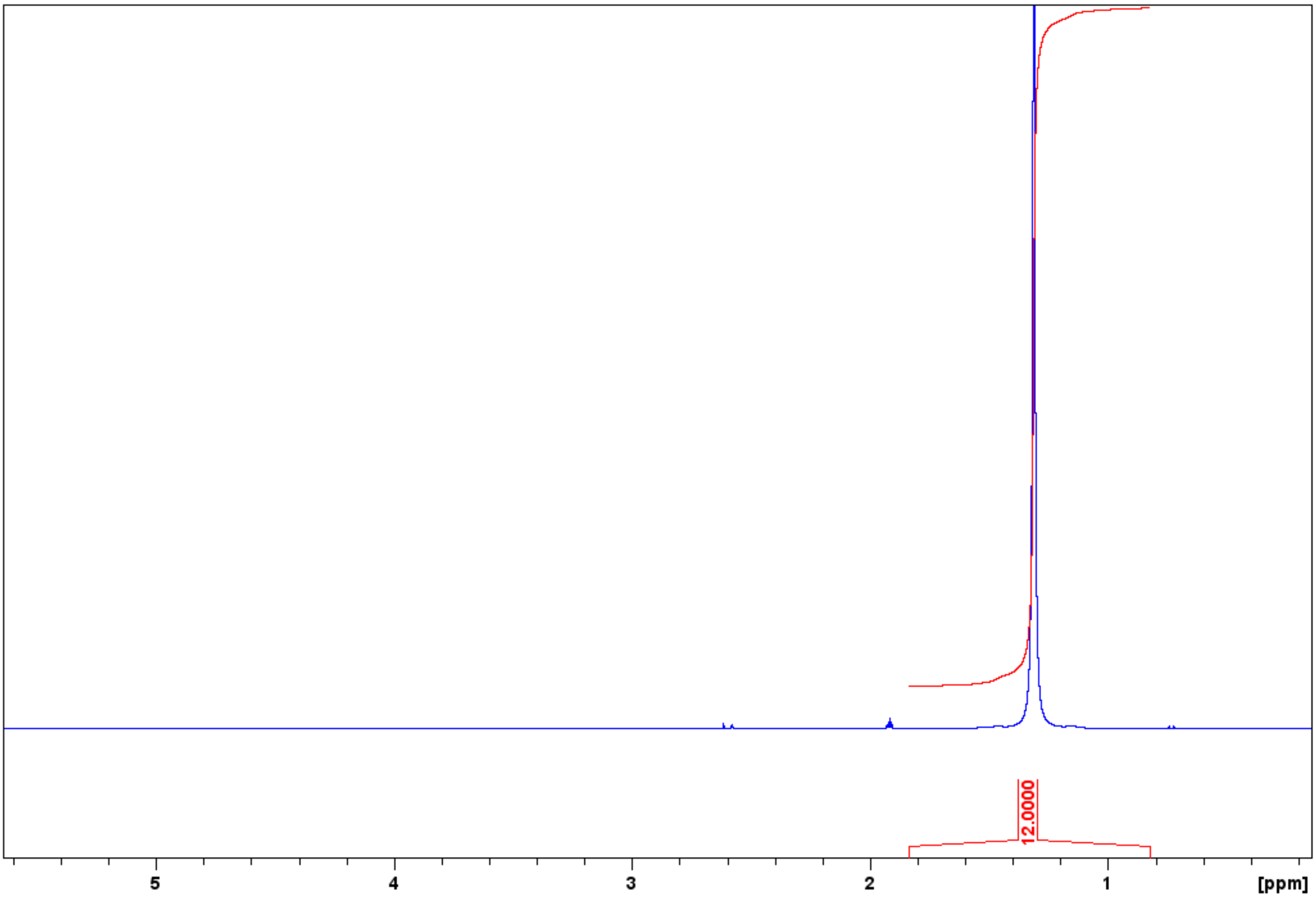

Figure S24. ^{1}H NMR spectrum of cyclohexane layer after mixing with 1,3-propanediol in acetone-d_6.
